# Overcoming Photochemical Limitations in Covalent Organic Frameworks: Low‐Energy Light Driven Selective ^1^O_2_ Generation Achieved by Donor–Acceptor Strategy

**DOI:** 10.1002/anie.202508078

**Published:** 2025-06-18

**Authors:** Jikuan Qiu, Hanping Zhai, Yuling Zhao, Yucheng Jin, Zhiyong Li, Huiyong Wang, Zhongping Li, Jianji Wang, Jong‐Beom Baek

**Affiliations:** ^1^ School of Chemistry and Chemical Engineering Key Laboratory of Green Chemical Media and Reactions Ministry of Education Henan Normal University 46 Jianshe Road E. Xinxiang Henan 453007 P.R. China; ^2^ Department of Energy and Chemical Engineering/Center for Dimension‐Controllable Organic Frameworks Ulsan National Institute of Science and Technology 50 UNIST‐gil, Eonyang‐eup, Ulsan Ulju‐gun 44919 Republic of Korea

**Keywords:** Covalent organic frameworks, Low‐energy light, Photocatalysis, Singlet oxygen

## Abstract

Singlet oxygen (^1^O_2_) plays a crucial role in various photocatalytic oxidation reactions; however, achieving high‐efficiency and selective ^1^O_2_ production under low‐energy light remains a challenge. Herein, we present a novel donor–acceptor (D–A) strategy in covalent organic frameworks (COFs) to regulate the localized electronic state structures for efficient and selective ^1^O_2_ generation under low‐energy light. Notably, the rationally incorporation of the negatively charged carbonyl groups into the basal plane of the COF strengthens the D–A interaction, improves light harvesting in the lower‐energy region, and facilitates highly selective ^1^O_2_ generation through a coupled charge‐transfer mechanism. As a result, the engineered COF demonstrates exceptional photocatalytic performance in ^1^O_2_ driven advanced oxidation, enabling gram‐scale production under red light, even when operating through translucent barriers. A mechanistic study revealed that the distinct ^1^O_2_ production under low‐energy light is attributed to the spatially locked structure and charge localization around active centers. These features enhance strong π–π stacking interaction, promote effective charge separation and transport properties, and ultimately facilitate the activation of O_2_ to ^1^O_2_. This study paves the way for the development of high‐performance COF photocatalysts for low‐energy light‐driven reactive oxygen species generation in advanced oxidation processes.

## Graphical abstract

We demonstrate for the first time that a novel donor–acceptor strategy in covalent organic frameworks (COFs) has been developed to regulate the localized electronic state structures for efficient and selective ^1^O_2_ generation under low‐energy light. The engineered COF selectively produced ^1^O_2_ under red light through a coupled charge transfer mechanism and exhibited exceptional photocatalytic performance in ^1^O_2_ driven advanced oxidation, achieving gram‐scale production under red light, even when operating through translucent barriers.

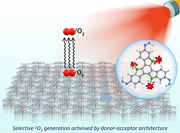

## Introduction

Light‐assisted oxidation reactions offer a sustainable strategy for converting solar energy into high‐value‐added chemicals. Among these reactions, singlet oxygen (^1^O_2_) is a widely used reactive oxygen species (ROS). Compared to other ROS, ^1^O_2_ with an unoccupied π* orbital demonstrates superior selectivity and heightened reactivity when interacting with organic matter, particularly in advanced oxidation processes (AOP).^[^ ^1^, ^2^, ^3^ ^]^ In recent years, researchers have enhanced the efficiency and selectivity of ^1^O_2_ generation by designing and synthesizing photocatalysts with specific structures, such as metal–nitrogen‐doped carbon, transition‐metal complexes, and metal–organic frameworks.^[^ ^4^, ^5^, ^6^, ^7^, ^8^ ^]^ These studies not only explore the mechanism of ^1^O_2_ generation but also confirm its potential applications in photocatalytic oxidation reactions.

Covalent organic frameworks (COFs) are an emerging class of porous organic polymers composed of periodic units connected by covalent bonds.^[^ ^9^, ^10^, ^11^, ^12^, ^13^, ^14^, ^15^ ^]^ With their well‐defined layered structures, COFs has demonstrated great potential for improving mass and charge transport pathways in photocatalytic oxidation reactions.^[^ ^16^, ^17^, ^18^, ^19^, ^20^, ^21^, ^22^, ^23^, ^24^, ^25^, ^26^, ^27^, ^28^ ^]^ Their tunable structure and functionality allow precise control over ROS generation through site engineering, enabling efficient and selective ^1^O_2_ production.^[^ ^8^, ^29^, ^30^, ^31^, ^32^, ^33^, ^34^, ^35^ ^]^ In most cases, ^1^O_2_ generation relies on high‐energy sources such as blue light or high‐power xenon lamps as most materials have narrow absorption ranges. The use of low‐energy light, such as deep red or near‐infrared light, remains rare^[^ ^36^, ^37^ ^]^ due to the short lifetime of the excited states that are obtained under low‐energy light excitation. However, high‐energy photons have inherent limitations, including poor penetration through reaction media, competitive absorption by reactant species, and limited solar spectrum coverage. Therefore, the development of photoactive COFs capable of harvesting low‐energy light for selective ^1^O_2_ generation is urgently required but remains highly challenging.

Charge localization, first proposed by the Nobel laureate Anderson,^[^ ^38^ ^]^ is a powerful method for controlling charge dynamics in semiconductor materials to maximize performances.^[^ ^39^, ^40^ ^]^ The behavior of localized electrons can significantly influences a material's response to light, enhancing the absorption of specific wavelengths of light and improving photoelectric conversion efficiency.^[^ ^41^ ^]^ Particularly, breaking the charge symmetry of an active center facilitate charge localization in semiconductor materials, significantly promoting space charge separation and enhancing O_2_ adsorption and activation, thereby boosting ROS production.^[^ ^42^ ^]^ Additionally, intermolecular interactions in 2D organic molecular crystals, particularly π–π stacking, play a crucial role in determining their physical and chemical properties. Strong π–π stacking interactions enhance thermal stability, high carrier mobility, and unique physical properties.^[^ ^43^, ^44^ ^]^ By integrating these advantages through strategic molecular design, it may be possible to overcome photochemical limitations in COFs, ultimately achieving the selective production of ROS under low‐energy light.

**Scheme 1:**
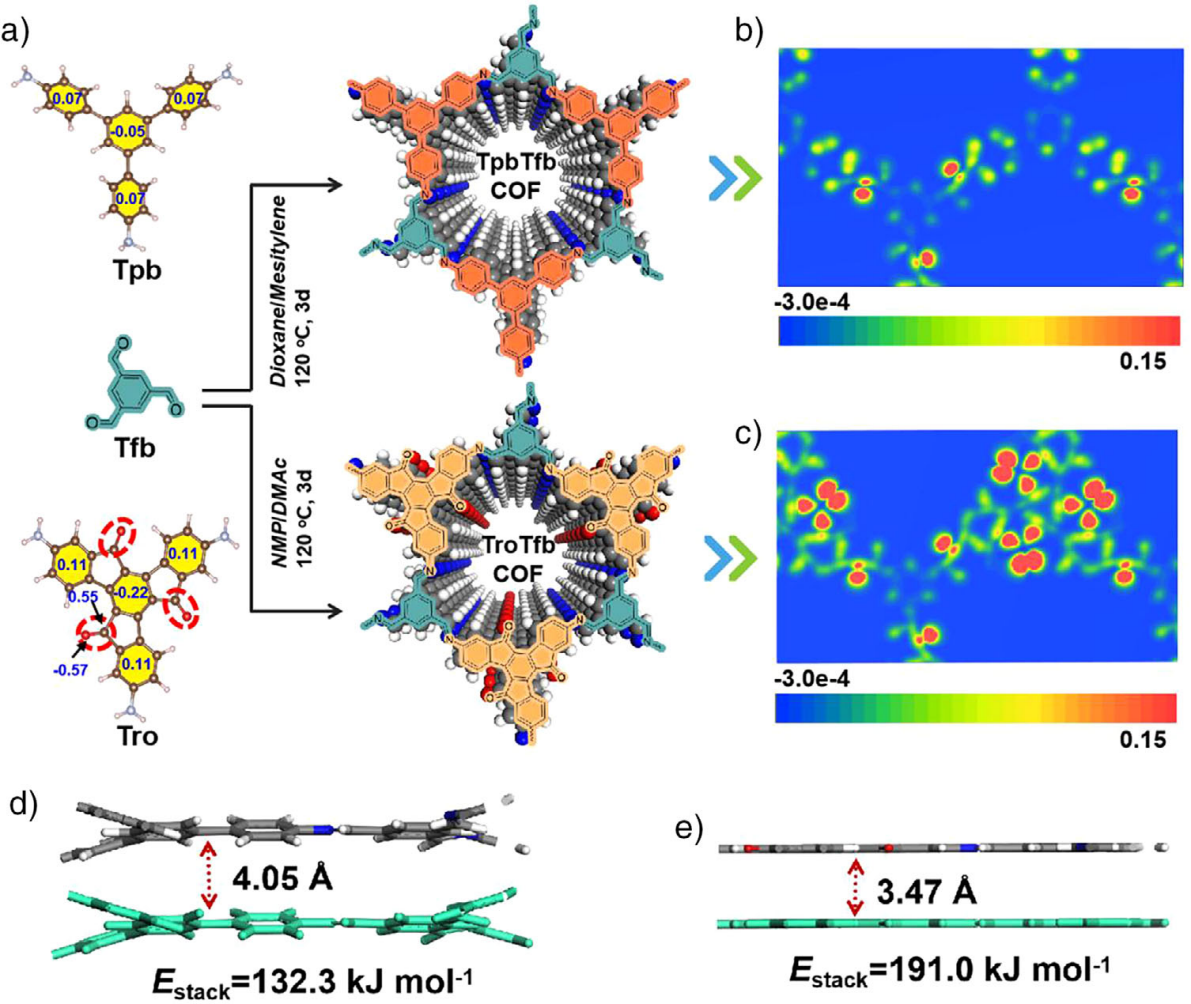
a) Synthetic scheme for TpbTfb‐COF and TroTfb‐COF. b) and c) Localized charge distribution of TpbTfb‐COF and TroTfb‐COF, respectively. d) and e) Adjacent layer packing of TpbTfb‐COF and TroTfb‐COF based on the eclipsed arrangements, respectively.

Herein, we develop a donor–acceptor (D–A) strategy in highly crystalline imine‐based COFs for the selective production of ^1^O_2_ under low‐energy light. By rationally incorporating the electron‐withdrawing carbonyl groups into two adjacent benzene rings of 1,3,5‐triphenylbenzene core, the resulting TroTfb‐COF exhibits enhanced D–A interactions within the framework. This design not only promotes charge localization within the framework but also enhances the interlayer π–π stacking by the formation of a locked‐in coplanar conformation (Scheme 1). As a result, TroTfb‐COF exhibits stronger capability for visible‐light absorption (200–750 nm) than the pure carbon molecular framework model TpbTfb‐COF, indicating its potential as a red‐light‐based photocatalyst. TroTfb‐COF selectively produces ^1^O_2_ under red light by a coupled charge transfer mechanism, with its photocatalytic activity improving 10‐fold compared to TpbTfb‐COF. Due to the excellent penetration of red light, TroTfb‐COF has for the first time achieved the gram‐scale synthesis performance in advanced oxidation reaction, even when operating through translucent barriers. Electronic property analysis and density‐functional theory (DFT) calculations revealed that the distinct ^1^O_2_ generation was attributed to the spatially locked structure and charge localization around active centers, which endow the strong π–π stacking interaction with effective charge separation and transport properties, thereby promoting the activation of O_2_ to ^1^O_2_. These promising results open new avenue toward the selective control of ROS generation based on the D–A architecture.

## Results and Discussion

We first selected molecules 1,3,5‐Tris (4‐aminophenyl)benzene (Tpb) and 5*H*‐tribenzo[*a,f,k*]trindene‐5,10,15‐trione (Tro) as the amine‐based building blocks. Both monomers can be integrated with all aldehyde‐based monomer to afford the D–A structures. By comparing the charges on the benzene rings of these two molecules (Scheme 1a), we observed that the introduction of the carbonyl group (C═O) shifts the electron cloud of the entire conjugated system toward the C═O. As a result, the oxygen and carbon atoms carry strong negative and positive charges, respectively. Moreover, the central benzene ring becomes more negatively charged, whereas the other three benzene rings acquire more positive charges. TpbTfb‐COF and TroTfb‐COF, which share a similar topological structure, were then synthesized between 1,3,5‐benzenetricarboxaldehyde (Tfb) and Tpb or Tro, respectively. This approach enhances the D–A interaction strength within the COF framework by incorporation of C═O groups, thereby causing in rapid charge transfer.

Next, we calculated the electron density surfaces of both COFs. Scheme 1b,c presents the localized scanning tunneling microscopy (STM) maps of the COFs skeleton. The higher electronic localization function value observed at the O═C‐ site indicates highly localized charge density. TroTfb‐COF exhibits a greater degree of localized electrons than TpbTfb‐COF, which contributes to further enhancing electron transfer.^[^ ^45^ ^]^ Furthermore, by examining the structural diagrams of the two COFs from both the front and side views reveals distinct spatial structures. The benzene rings cores in TroTfb‐COF adopt a more planar conformation than the propeller‐like TpbTfb‐COF (torsion angles 26.3° and 29.1°) (Figures S1 and S2). To evaluate the interlayer interactions, we employed density functional theory (DFT) calculations to determine the total π–π stacking energy per crystal cell bilayer (*E*_stack_). The calculated *E*_stack_ values were 132.3 kJmol for TpbTfb‐COF and 191.0 kJmol for TroTfb‐COF (Scheme 1d,e). This enhancement in stacking energy is attributed to the incorporation of C═O groups in two adjacent benzene rings, which locks these rings onto the same plane, thereby significantly strengthening the intermolecular π–π stacking interactions in the COF.^[^ ^46^ ^]^ Consequently, these structural modifications enhance light absorption and catalytic efficiency for activating oxygen molecules to generate specific ROS species.

**Figure 1:**
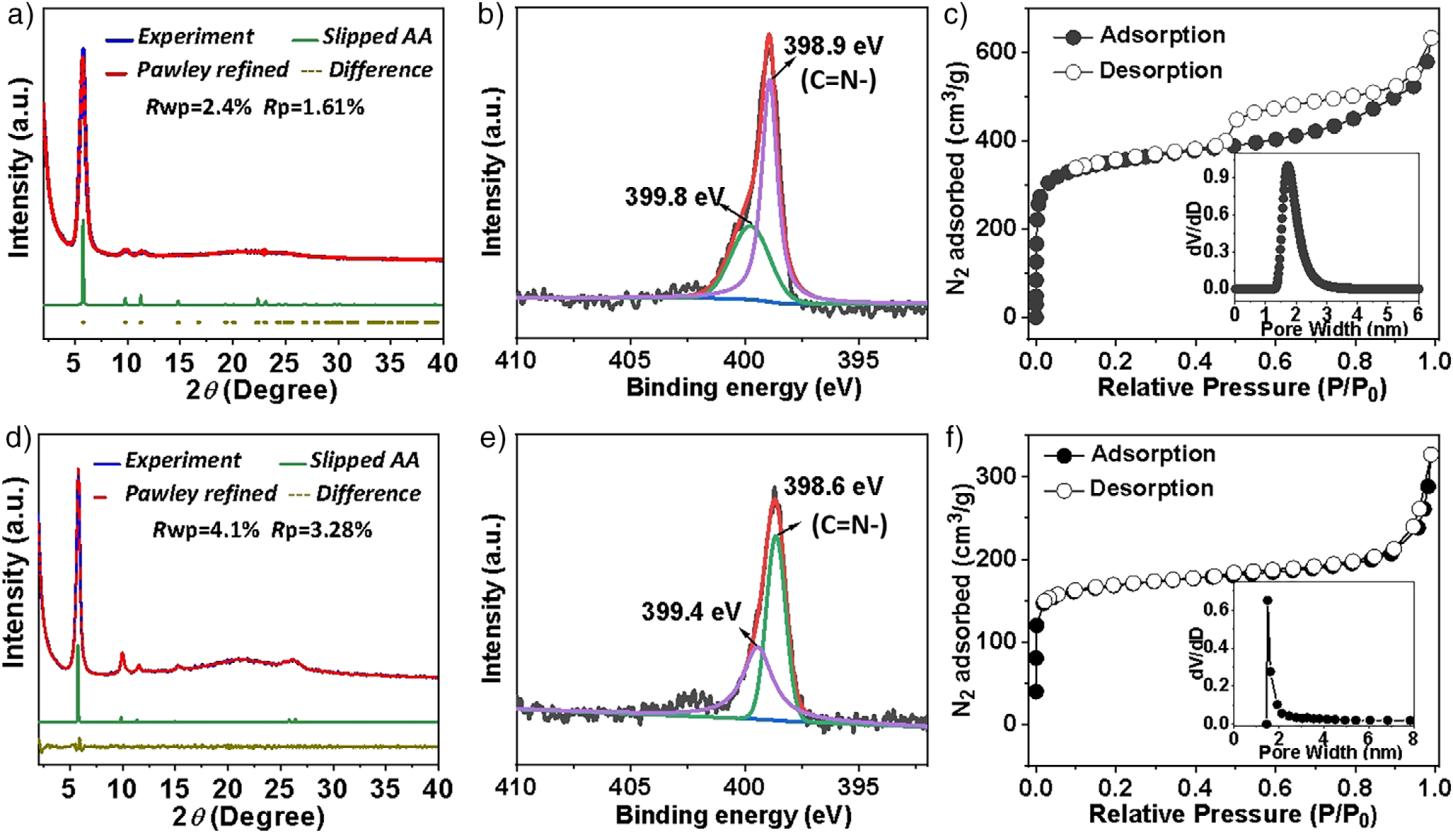
Characterization of TpbTfb‐COF and TroTfb‐COF. a) and d) Experimental and simulated PXRD patterns (AA stacking) as well as the different curves of TpbTfb‐COF and TroTfb‐COF, respectively. b) and e) High‐resolution XPS spectrum of N 1s for TpbTfb‐COF and TroTfb‐COF, respectively. c) and f) Nitrogen adsorption isotherm profiles of TpbTfb‐COF and TroTfb‐COF, respectively, collected at 77 K. Inset, the pore size distribution profiles.

TpbTfb‐COF and TroTfb‐COF were synthesized using a solvothermal method. The synthesis of TroTfb‐COF was carried out in a solvent mixture (3.3 mL, V/V/V = 5: 5: 1) of *N*‐methyl‐2‐pyrrolidone (NMP), *N,N*‐dimethy‐lacetamide (DMAc), and acetic acid (6 M) under solvothermal conditions at 120 °C for 72 h (refer to the Experimental Section in Supporting Information for details). Fourier transform infrared (FTIR) spectroscopy confirmed the successful condensation and formation of imine linkages through condensation. The FTIR spectra of both COFs exhibited strong absorption peaks at ∼1573 cm^−1^, corresponding to ‐C═N─ linkages (Figures S3 and S4). Additionally, the disappearance of aldehyde group (*ν*_HC_═_O_ ≈ 1695 cm^−1^) and amine group (*ν*_N─H_ *≈* 3300 cm^−1^) peaks further confirmed the complete formation of imine bonds via the Schiff‐base reaction.^[^ ^47^, ^48^, ^49^ ^]^ X‐ray photoelectron spectroscopy (XPS) also validated the formation of the imine structures, with the N 1s peaks at 398.9 and 398.6 eV assigned to the C═N fragment of TpbTfb‐COF and TroTfb‐COF, respectively (Figures 1b,e, S5, and S6).^[^ ^50^, ^51^, ^52^ ^]^ The formation of imine linkages was further confirmed by solid‐state ^13^C NMR spectra (Figures S7 and S8), which exhibited characteristic peaks at ∼155 ppm corresponding to the C═N bonds. Scanning electron microscopy (SEM) and transmission electron microscopy (TEM) reveal that both COFs exhibit a unique rod‐like morphology composed of self‐assembled nanometer‐scale crystallites (Figures S9–S12).

The crystallinity of TpbTfb‐COF and TroTfb‐COF was analyzed using powder X‐ray diffraction (PXRD). As shown in Figure 1a,d, the PXRD profiles of both COFs displayed a prominent reflection in the low‐angel region at 2θ ≈ 5.3°, corresponding to the (100) facet of a hexagonal lattice. Additional weaker reflections at ∼10.7° and ∼14.1° were assigned to the (200) and (210) facets, respectively, confirming the formation of a high crystalline and ordered framework structure. The experimental PXRD patterns closely matched the simulated patterns generated for an eclipsed AA stacking mode using Material Studio (Figures 1a,d, S13, and S14). After Pawley refinement, TpbTfb‐COF is assigned to triclinic space group (*P*1) with the unit cell parameters *a* = 18.71 Å, *b* = 18.75 Å, *c* = 4.03 Å, whereas TroTfb‐COF is assigned to hexagonal space group (*P*6) with the unit cell parameters *a* = *b* = 18.35 Å, *c* = 3.48 Å (Tables S1 and S2). Furthermore, the refined PXRD patterns agreed well with the experimental PXRD patterns with low‐profile and weighted‐profile *R* values of 1.61% and 2.4%, respectively, for TpbTfb‐COF, and 3.28% and 4.1%, respectively, for TpbTfb‐COF, suggesting that both COFs adopted the AA stacking model.

The porosities of TpbTfb‐COF and TroTfb‐COF were determined using N_2_ adsorption–desorption isotherms at 77 K. Both COFs exhibited a reversible type‐I adsorption isotherm, a typical characteristic of microporous materials (Figure 1c,f). The calculated Brunauer–Emmett–Teller specific surface areas of TpbTfb‐COF and TroTfb‐COF were to be 1338 and 527 m^2^ g^−1^, respectively. Nonlocal density functional theory (NLDFT) analysis showed a narrow pore size distribution of 1.4 nm for TpbTfb‐COF and 1.8 nm for TroTfb‐COF, which agreed well with their theoretical values. Additionally, the chemical stability of the newly prepared COFs was evaluated by soaking them in various media. For example, TroTfb‐COF retained its crystallinity with negligible changes after treatment with 3 M NaOH, 3 M HCl solutions, THF, and methanol (Figure S15). However, TpbTfb‐COF almost lost its crystallinity under similar conditions (Figure S16), indicating lower stability than TroTfb‐COF. The high chemical stability of TroTfb‐COF could be attributed to the strong interlayer π–π stacking enabled by its highly planar conjugated framework, which not only reinforced the overall framework integrity but also reduced the accessibility of imine‐linkage to external chemical agents.

**Figure 2:**
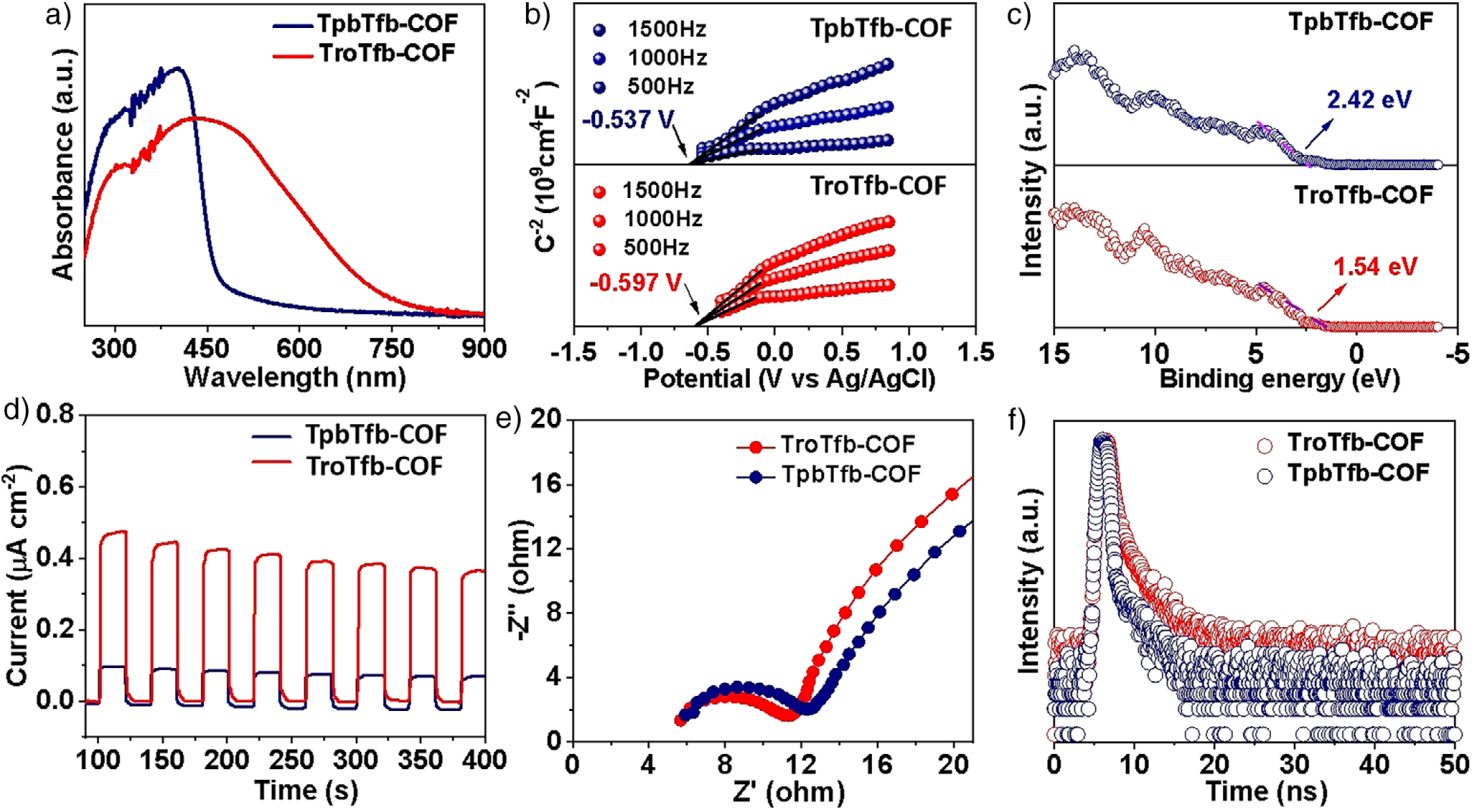
a) UV–visible diffuse reflectance spectra, b) Mott–Schottky curves, c) XPS‐VB spectra, d) photocurrent responses, e) EIS Nyquist plots, and f) TCSPC spectroscopy of each COF.

The photophysical properties of TpbTfb‐COF and TroTfb‐COF were investigated using UV–visible (UV–vis) spectroscopy. TroTfb‐COF exhibited significantly improved light absorption properties over its pure carbon molecular framework model TpbTfb‐COF (Figure 2a). TroTfb‐COF displayed broad absorption peaks in the 200—750 nm range, indicating good visible and near‐infrared light‐harvesting ability. Accordingly, the optical energy bandgaps (*E*_g_), calculated using the Tauc plot, were 2.76 and 1.91 eV for TpbTfb‐COF and TroTfb‐COF, respectively (Figures S17 and S18). Obviously, the *E*_g_ of TroTfb‐COF narrowed with increasing charge localization in the framework, which significantly improved its electrical performance. The narrow *E*_g_ indicated that TroTfb‐COF had high solar energy utilization efficiency and could be photoexcited at a higher wavelength akin to that of low‐energy light, such as red light. The broader light absorption range and narrower *E*_g_ of TroTfb‐COF could be attributed to the intrinsic nature of the symmetric carbonyl group linked conjugation. Additionally, the Mott–Schottky plot for TpbTfb‐COF and TroTfb‐COF showed a positive slope, a typical characteristic of an n‐type semiconductor (Figures 2b, S19, and S20). From the Mott–Schottky plots, the conduction band (CB) levels of TpbTfb‐COF and TroTfb‐COF were determined to be −0.537 and −0.597 V versus the Ag/AgCl electrode (−0.34 and −0.40 V versus the normal hydrogen electrode, NHE, pH 6.8). Clearly, the CB potential of all these COFs was sufficiently negative for the photocatalytic reduction of O_2_ to O_2_•^−^ (−0.33 V versus NHE). Subsequently, the valence band (VB) values were calculated to be 2.42 and 1.54 eV (versus NHE) for TpbTfb‐COF and TroTfb‐COF, respectively, which were consistent with the results obtained from the valence band XPS spectrum (XPS‐VB, Figure 2c). These findings reveal a significant enhancement in terms of photophysical properties due to the charge localization in the COFs achieved through the D–A architecture.

The charge separation efficiency was analyzed using transient photocurrent density and electrochemical impedance spectroscopy (EIS). Anodic photocurrents were tested under chopped red light (on/off) irradiation for all of the COFs. The photocurrent intensity of TroTfb‐COF was significantly higher than that of TpbTfb‐COF (Figure 2d), indicating greater charge separation efficiency in photocatalytic processes. As seen in the EIS test (Figure 2e), TroTfb‐COF exhibited a smaller semicircle radius than TpbTfb‐COF, suggesting lower impedance and interfacial charge transfer barriers, thereby facilitating a faster charge migration rate. Moreover, electron‐hole recombination ability was investigated using photoluminescence (PL) spectroscopy (Figure S21). Clearly, TroTfb‐COF exhibited the higher fluorescence intensity than that of the TpbTfb‐COF, demonstrating the strong suppression of radiative exciton recombination. Additionally, TroTfb‐COF showed a longer decay lifetime (0.91 ns), as determined by time‐correlated single photon counting (TCSPC) spectroscopy (Figure 2f), which was more than 10 times that of TpbTfb‐COF (0.087 ns).

**Figure 3:**
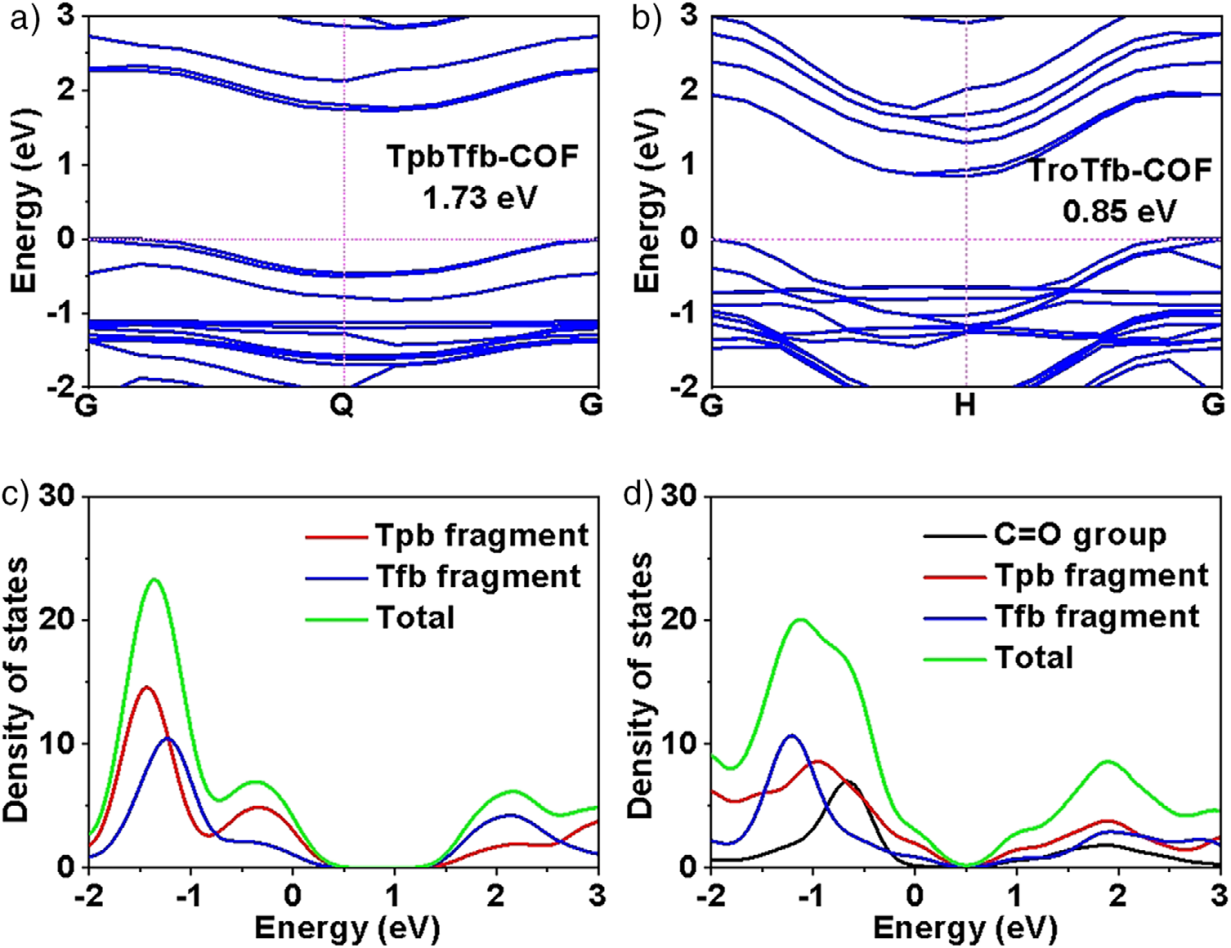
a) and b) Calculated electronic band structures of TpbTfb‐COF and TroTfb‐COF, respectively. Both structures exhibit indirect band gaps. Projected density of states (DOS) of c) TpbTfb‐COF and d) TroTfb‐COF. In Figure 3c, the contributions from the Tpb and Tfb fragments are shown. In Figure 3d, the Tro unit was decomposed into the Tpb fragment and the C═O group to better highlight their respective electronic contributions, along with the Tfb fragment. The total DOS is also presented in both cases for comparison.

To further explore the effect of charge localization in the benzene‐ring center, the electronic structures of both COFs were studied by theoretical calculations. The projected density of states (DOS) analysis suggested that TroTfb‐COF exhibited a lower indirect bandgap than TpbTfb‐COF (0.85 eV versus 1.73 eV) (Figure 3a,b), indicating a higher probability of electrons excitation in TroTfb‐COF. As revealed by the DOS and molecular structure decomposition analysis (Figures 3c,d and S22), the introduction of the C═O group significantly enhanced the DOS intensity of the valence band compared to that in TpbTfb‐COF, demonstrating that the primary contributions to the electronic structure originated from C═O. As a result, a greater number of electrons in TroTfb‐COF could be excited to the conduction band under light irradiation, facilitating efficient electron transfer. These results indicate that the charge localization effect in the skeleton could enhance the charge separation and transport properties upon red light irradiation.

**Figure 4:**
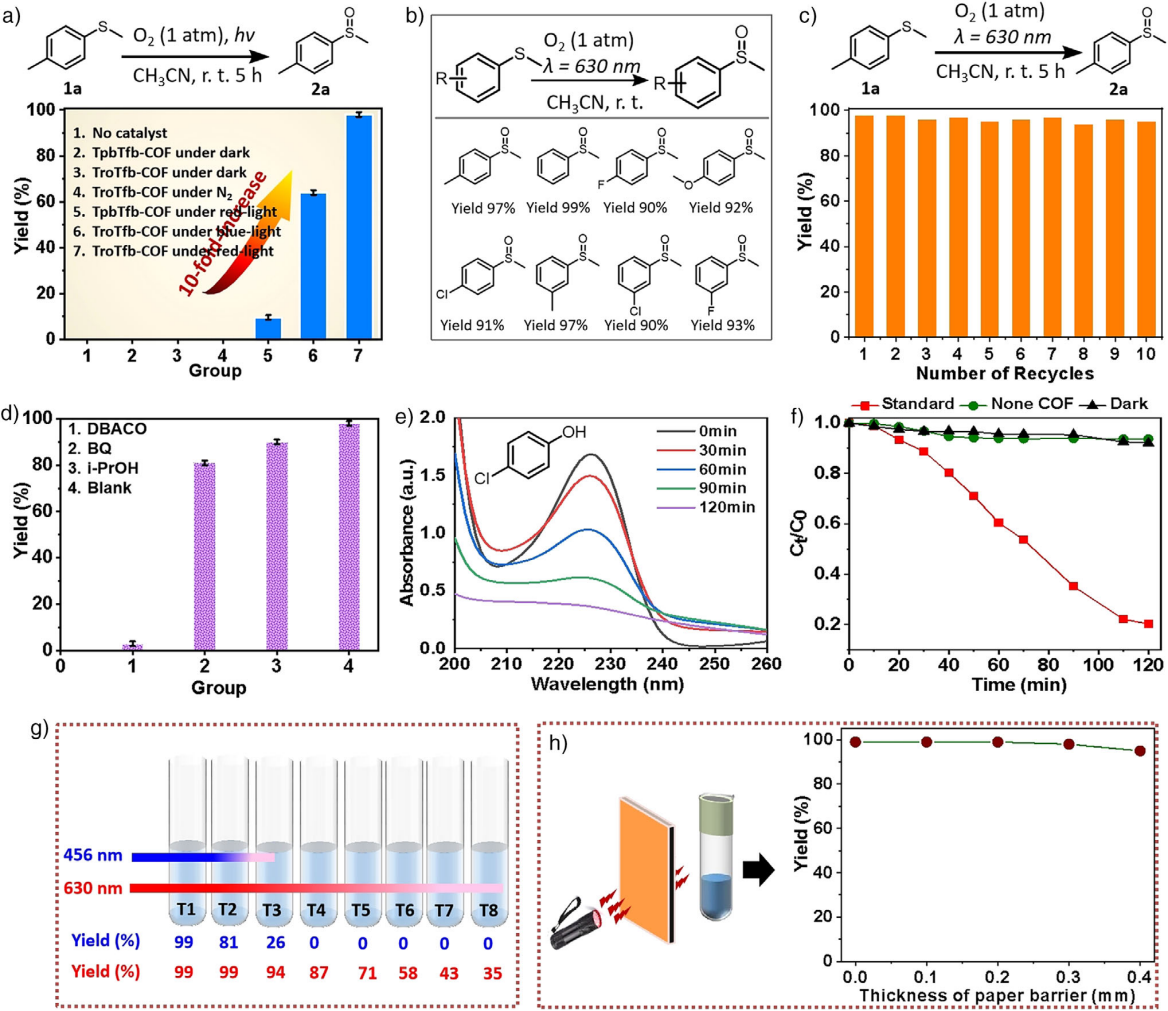
a) Yields of the product under different reaction conditions when TroTfb‐COF and TpbTfb‐COF were used. b) Substrate scope for oxidation of sulfides using TroTfb‐COF as photocatalyst. c) Recyclability of TroTfb‐COF in the photocatalytic oxidation of sulfides. d) Yields of products obtained during photocatalytic oxidation of sulfides in the presence of different scavengers and TroTfb‐COF. e) Time‐dependent UV–vis absorption spectra recording oxidation degradation of 4‐chlorophenol (4‐CP) over TroTfb‐COF. f) Degradation of 4‐CP by TroTfb‐COF under different condition. g) Yields of the products obtained during the photocatalytic oxidation of thioanisole in different reaction tubes using different light source. h) Photocatalytic oxidation of thioanisole in the presence of a paper barrier (left) and the dependence of product yield after red light irradiation for 5 h on the thickness of the paper (right).

To examine the photocatalytic performance of TpbTfb‐COF and TroTfb‐COF, the oxidation of organic sulfides, which is mediated by ^1^O_2_ and can selectively convert thioanisole to sulfoxide,^[^ ^27^ ^]^ has been studied under red light irradiation (*λ* = 630 nm) (Figure 4a). The reaction proceeded smoothly in an O_2_ environment using TroTfb‐COF as the photocatalyst. For comparison, the photocatalytic performances of TroTfb‐COF and TpbTfb‐COF were evaluated through seven different experiments. The product yield was 0% in the absence of the catalyst. Similarly, no reaction occurred without visible light irradiation, even when TroTfb‐COF or TpbTfb‐COF was used as catalysts. These results indicated that both a catalyst and light were essential for the oxidation process. Furthermore, TroTfb‐COF did not drive the reaction under an N_2_ atmosphere despite red light irradiation, confirming that O_2_ played a crucial role. The catalytic efficiencies of TroTfb‐COF and TpbTfb‐COF were then compared under standard conditions. The yield for the TpbTfb‐COF catalyzed reaction was only 10%, whereas the TroTfb‐COF catalyzed‐reaction achieved a remarkably high yield of 98%, demonstrating that TroTfb‐COF exhibited a 10‐fold increase in photocatalytic efficiency compared to TpbTfb‐COF. Notably, TroTfb‐COF showed a higher catalytic performance under the low‐energy red irradiation than under a high‐energy blue source, which was probably due to its excellent charge transfer and separation efficiency of the photogenerated electron‐hole pairs in the presence of the red light.

The reaction scope was further investigated using TroTfb‐COF as the catalyst. As shown in Figure 4b, various aryl sulfides bearing fluorine, methoxy, or methyl functional groups were selected to serve as the substrates. TroTfb‐COF exhibited excellent photocatalytic activity with all sulfides selectively oxidized to sulfoxides in high yields (90%–99%) in CH_3_CN under red light. This result indicated the efficient production of ^1^O_2_.

To assess recyclability, the catalyst was consecutively used 10 times. TroTfb‐COF maintained excellent catalytic performance and stability even after the 10th cycle (Figure 4c). PXRD and FTIR analysis of TroTfb‐COF after the catalytic experiments confirmed that its structural integrity was well preserved (Figures S23 and S24). The feasibility of the larger‐scale product preparation was explored using 0.02 mol of thioanisole under identical reaction conditions, yielding 90% of the product (2.06 g). This is the first reported instance of gram‐level sulfoxide synthesis using TroTfb‐COF as a red‐light‐based photocatalyst. The successful large‐scale synthesis can be attributed to the robust nature of TroTfb‐COF and its strong absorption at 630 nm, which prevents multiple detrimental reaction pathways.

Scavenging tests were conducted to confirm that ^1^O_2_ was the primary ROS involved in the oxidation of thioanisole using TroTfb‐COF (Figure 4d). The introduction of the hydroxyl radical (**^.^** OH) scavenger isopropanol (iPrOH) had minimal impact on the reaction yielding 89% yield. Similarly, the use of *p*‐benzoquinone (BQ) as a quencher for superoxide radical (O_2_•^−^) only slightly affected the reaction. However, when DBACO was introduced into the system as a ^1^O_2_ scavenger, the reaction yield drastically decreased to 3%. These findings revealed that O_2_ was primarily activated to ^1^O_2_, and the active oxygen species played an indispensable role in the photocatalytic reaction.

One of the most remarkable features of red light is its minimal absorption and high penetration, enabling its use in a wide range of medicine applications for both diagnosis and treatment.^[^ ^53^, ^54^ ^]^ Next, we compared the reaction performance under red light (*λ* = 630 nm) and white light (*λ* = 456 nm) irradiation using the oxidation of thioanisole as a model reaction. In this experiment, eight reaction tubes were arranged in a row, with the irradiation light applied from the left side only (Figure 4g). The results showed that the 456 nm lamp produced a high yield (99%) in the first tube; however the yield dropped to 26% in the third tube. No reactions occurred in tubes four through eight due to the limited penetration of 456 nm light. However, the 630 nm lamp achieved a 99% yield in the first and second tubes and maintained respectable yields (94%–71%) in the third, fourth, and fifth tubes. The yield in the final tube was 35%. To further demonstrate its penetration ability, the red‐light‐driven oxidation of sulfides was conducted in a vessel screened by a translucent barrier, such as a sheet of A4 paper with a thickness of approximately 0.1 mm (Figure 4h, left). The results showed that a 99% yield was obtained under red light illumination screened by a single sheet of paper (Figure 4h, right). When the A4 paper thickness was increased to four sheets, a high yield (97%) was still achieved, highlighting the strong tissue penetration ability of red light and the excellent photocatalytic performance of the catalyst.

Such an extraordinarily high photocatalytic oxidation activity of TroTfb‐COF prompted us to explore other red‐light‐driven oxidation reactions. First, we investigated the oxidative degradation performance of 4‐chlorophenol (4‐CP), a common toxic and poorly biodegradable phenolic organic pollutant in water. Under the red light illumination, 4‐CP was completely degraded by TroTfb‐COF within 3 h (Figure 4e). Control experiments demonstrated that in the absence of either light or catalyst, only approximately 5% of 4‐CP was removed (Figure 4f), confirming the essential role of both light illumination and the catalyst in the degradation process. The degradation products were analyzed using GCMS (Figures S25–S29), yielding results consistent with previous reports.^[^ ^55^ ^]^

Furthermore, the formation of benzimidazole heterocycles via the cascade reaction between *o*‐phenylenediamine and benzaldehyde, mediated by ROS,^[^ ^56^ ^]^ was studied under red light irradiation. The reaction proceeded smoothly in a protonic solvent in an O_2_ environment using TroTfb‐COF as the photocatalyst (Table S3). A series of *o*‐phenylenediamine and benzaldehyde derivatives reacted smoothly, converting into 5‐membered benzimidazoles (Table 1). Both electron‐donating and electron‐withdrawing groups at the *p*‐position of benzaldehyde were well tolerated, yielding the desired products **3a–3f** in excellent yields (90%–98%). Interestingly, when the aromatic aldehyde was replaced with alkyl or heterocyclic aldehydes, they also reacted with *o*‐phenylenediamine to furnish products **3g–3i** in a high yield. In addition, the reaction between aromatic aldehydes and 2‐aminobenzam was explored to synthesize 6‐membered benzquinazolines, aiming to evaluate the synthetic potential of low‐energy red light for photochemical transformations. Under red light irradiation, moderate yields of **4a–d** were obtained likely due to the low activity of the ─NH_2_ group in 2‐aminobenzamide within the reaction system.

**Table 1:** The model reaction and substrate scope for synthesis of five‐ and six‐membered nitrogen heterocycles using TroTfb‐COF as photocatalyst.^a)^

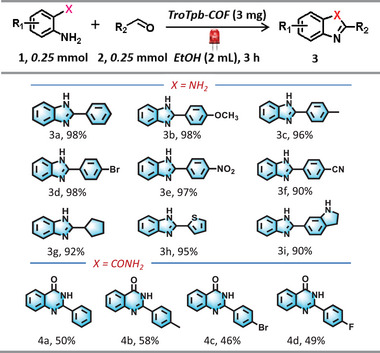

^a)^ Using 1,3,5‐trimethoxybenzene as an internal standard, yield was monitored by ^1^H NMR.

**Figure 5:**
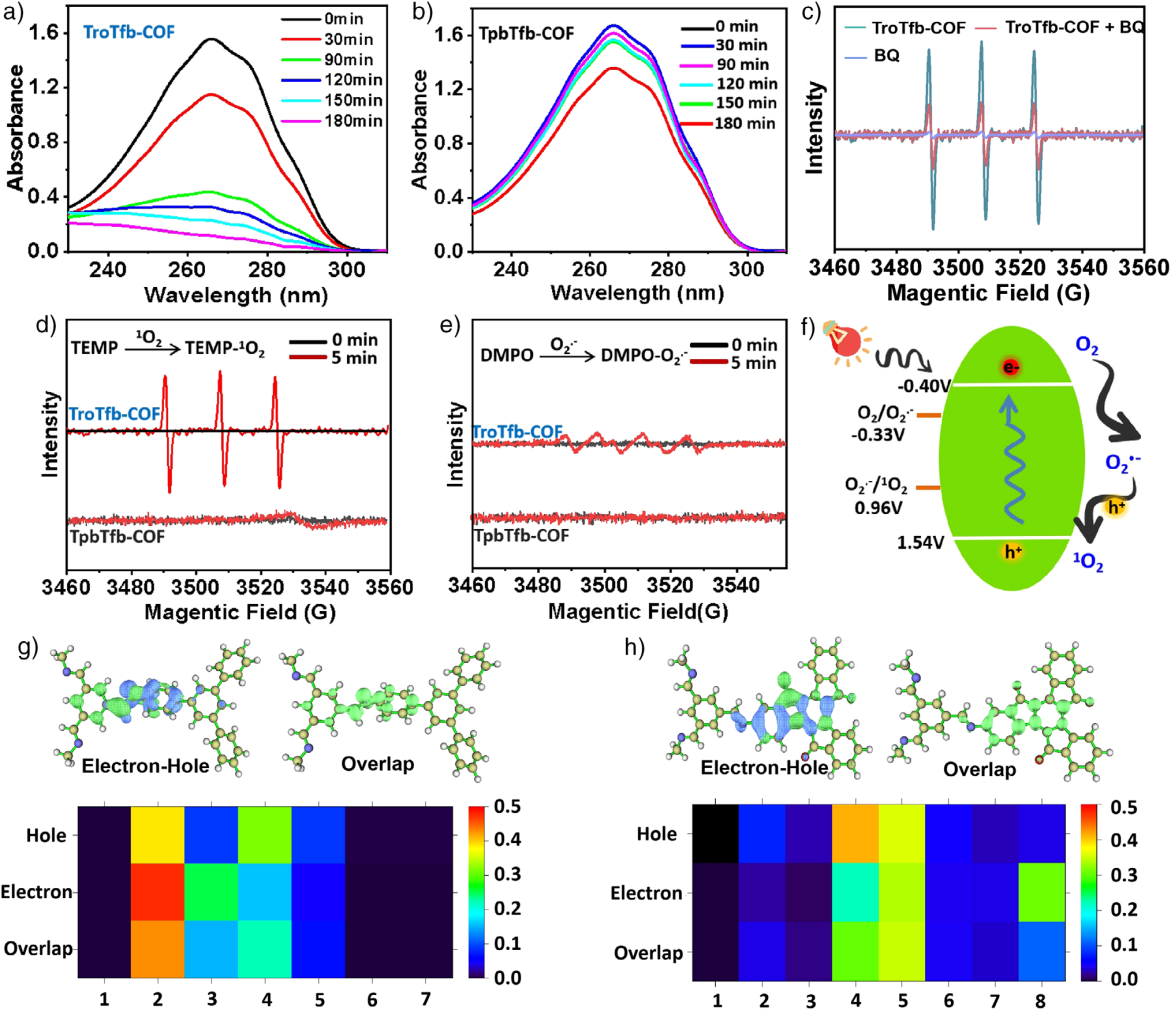
UV–vis spectra of the α‐terpinene solution with a) TroTfb‐COF and b) TpbTfb‐COF in O_2_ atmosphere under red light irradiation. c) ESR trapping experiments of TEMP‐^1^O_2_ with added BQ. ESR spectra of d) TroTfb‐COF and TpbTfb‐COF in the presence of TEMP and e) TroTfb‐COF and TpbTfb‐COF in the presence of DMPO, in O_2_ atmosphere under red light irradiation. The signal amplitudes have been corrected based on the instrument's internal calibration to allow for quantitative comparison. f) Proposed mechanism of O_2_ activation on TroTfb‐COF. Electron‐hole (green, electron; blue, hole) and the overlaps distribution of g) TpbTfb‐COF and h) TroTfb‐COF. The heat maps below show the localization probabilities of holes, electrons, and their overlap along different specific structural fragments of the COF framework. Warmer colors (closer to red) indicate higher probability density.

To clarify the specific ^1^O_2_ produced by TroTfb‐COF, a series of the oxidation reactions of α‐terpinene to ascaridole were carried out. The extent of α‐terpinene oxidation can be assessed by monitoring the increasing absorbance peak at ∼270 nm. As depicted in Figure 5a,b, TroTfb‐COF efficiently oxidized α‐terpinene into the corresponding peroxide under red light irradiation within 3 h, whereas TpbTfb‐COF exhibited weaker oxidation performance under the similar conditions, highlighting the significantly greater ability of the former in the ^1^O_2_ production. Furthermore, nitro blue tetrazolium (NBT), which can be reduced solely by O_2_^•−^, was tested with TroTfb‐COF. The time‐resolved absorption spectra of NBT at 260 nm showed negligible changes in absorbance with prolonged irradiation time (Figure S31), indicating the absence of O_2_^•−^ generation. Additionally, terephthalic acid (TA) was employed as a probe to detect ^•^OH radicals. The absorption spectra of TA at 240 nm exhibited only minimal changes (Figure S32), suggesting an insignificant ^•^OH signal. These findings indicate that TroTfb‐COF effectively and selectively activates O_2_ molecules to generate ^1^O_2_ rather than O_2_^•−^ or ^•^OH. Then, the yields of ROSs were quantitatively determined for TroTfb‐COF based on these spectral data (Figures S30–S32).^[^ ^32^, ^36^ ^]^ It was found that the yields was 10.6 µM·s^−1^ for ^1^O_2_, 0.61 µM·s^−1^ for O_2_^•−^, and 0.53 µM·s^−1^ for ^•^OH, respectively. From these results, the calculated selectivity for ^1^O_2_ was 90%. In addition, TroTfb‐COF also exhibited superior ^1^O_2_ production performance compared to the majority of reported metal‐free and even some metal‐based photocatalysts (Table S4), highlighting the effectiveness of TroTfb‐COF as a high‐performance photocatalyst for ^1^O_2_ generation under red light.

Electron spin resonance (ESR) spectroscopy was further employed to distinguish the specific ROS generation by TroTfb‐COF using ^1^O_2_ and O_2_^•−^ trapping agents. 2,2,6,6‐Tetramethylpiperidine (TEMP) was used as a ^1^O_2_ trapping agent, which converts to the TEMPO upon capturing ^1^O_2_. Similarly, 5,5‐dimethyl‐1‐pyrroline‐*N*‐oxide (DMPO) served as an O_2_^•−^ trapping agent, forming the stable DMPO‐O_2_^•−^ complex.^[^ ^28^ ^]^ A distinct characteristic signal of the TEMPO‐^1^O_2_ adduct was observed in the TroTfb‐COF system, conforming ^1^O_2_ production upon light irradiation (Figure 5d). In contrast, only a weak signal of the DMPO‐O_2_^•−^ adduct was detected (Figure 5e). The intensity of the TEMPO‐^1^O_2_ signal was more than five times higher than that of DMPO‐O_2_^•−^. In addition, no DMPO‐•OH signals were observed (Figure S33), indicating that O_2_ was primarily activated into ^1^O_2_ rather than other ROS. However, no radical ROSs signals were detected in the TpbTfb‐COF system after red light irradiation. This result could be attributed to its narrow absorption ranges, and the electrons in the valence band of TpbTfb‐COF could not be excited and migrate to the conduct band under red light irradiation.

The conduction band potential of TroTfb‐COF is higher than the oxidation potential of water, indicating that OH^.^ could not be generated. Additionally, the conduction band potential of TroTfb‐COF is lower than the reduction potential of O_2_ (O_2_/O_2_^•−^, −0.33 V versus NHE), suggesting that the single electron reduction of O_2_ to O_2_^•−^ is thermodynamically favorable. When TroTfb‐COF is excited by red light, the electrons are transferred to O_2_ molecules adsorbed on the carbonyl site to afford O_2_^•−^, and the generated O_2_^•−^ is then oxidized to ^1^O_2_ by the holes of TroTfb‐COF (O_2_^•−^/^1^O_2_, 0.96 V versus NHE). O_2_ can also convert into other active species such as HO_2_^−^ and H_2_O_2_ by protonation and dismutation. However, the reaction is completely inhibited in the absence of O_2_, conforming that ROS play a dominant role in TroTfb‐COF mediated photocatalysis. To clarify the source of producing ^1^O_2_, the ESR trapping experiments of TEMP‐^1^O_2_ for TroTfb‐COF with added BQ was investigated. As depicted in Figure 5c, the significant reduction in the TEMP‐^1^O_2_ signal suggests that the generated ¹O_2_ originates from O_2_^•−^. Furthermore, hole quencher KI and electron quencher DDQ were added into the sulfoxidation system, which can inhibit the electron transfer pathway and further suppress the formation of O_2_^•−^. It was found that the yields significantly reduced from 98% to 36% and 11%, respectively (Table S5), indicating that holes and electrons play an important role in the photocatalytic molecular oxygen activation, which the holes will react with O_2_^•−^ to form the ^1^O_2_.^[^ ^57^, ^58^ ^]^ Based on these findings, we conclude that the ^1^O_2_ is generated through coupled charge transfer mechanism rather than energy transfer (Figure 5f).

TD‐DFT calculations were performed to investigate the hole‐electron distribution within the framework. Here, we choose the S0→S1 transition to study electron excitation characteristics. As shown in Figure 5g,h, the spatial distributions of electrons, holes, and their overlap are visualized for TpbTfb‐COF and TroTfb‐COF, respectively. The heat maps below the orbital diagrams represent the localization probability across different repeating units of the COF structures, labeled as regions 1–7 (G) and 1–8 (H). These segments correspond to specific structural fragments, as illustrated in Figures S34 and S35. The holes and electrons of TpbTfb‐COF were primarily distributed in the C═N bonds, whereas the holes in TroTfb‐COF were concentrated in the central region of the benzene ring containing C═O groups. This result indicates that the oxidation active sites are primarily located in the C═O moiety. In addition, TroTfb‐COF exhibited a higher hole density than TpbTfb‐COF, enhancing redox reaction efficiency. These results demonstrate that adsorbed O_2_ molecules in TroTfb‐COF facilitate efficient charge transfer, thereby triggering ^1^O_2_ production and significantly boosting photocatalytic oxidation performance.

## Conclusion

In conclusion, we developed an efficient D–A strategy for selective ^1^O_2_ generation using a COF photocatalyst under low‐energy light. By rationally introducing negatively charged carbonyl groups into the basal plane of the COF, the designed TroTfb‐COF, which strengthens the D–A interaction within the framework, demonstrated enhanced charge separation efficiency and a broader absorption wavelength range than the pure carbon molecular framework model TpbTfb‐COF. Remarkably, TroTfb‐COF selectively produced ^1^O_2_ under red light through a coupled charge transfer mechanism, exhibiting a 10‐fold increase in photocatalytic performance compared to TpbTfb‐COF. More importantly, owing to the excellent penetration of red light, TroTfb‐COF has for the first time achieved gram‐scale synthesis performance in advanced oxidation reaction under red light, even when operating through translucent barriers. Mechanistic studies revealed that the distinct ^1^O_2_ production under low‐energy light was attributed to the spatially locked structure and charge localization around active centers in COFs. These structural features facilitated strong π–π stacking interactions and effective charge separation and transport, thereby promoting the activation of O_2_ to ^1^O_2_. This study not only structurally enriches the family of red‐light‐based photocatalysts but also provides valuable insights into the selective control of ROS in advanced oxidation through a D–A strategy.

## Conflict of Interests

The authors declare no conflict of interest.

## Supporting information



Supporting Information

## Data Availability

The data that support the findings of this study are available from the corresponding author upon reasonable request.
