# Supplementary material for: Overcoming Photochemical Limitations in Covalent Organic Frameworks: Low‐Energy Light Driven Selective 1O2 Generation Achieved by Donor–Acceptor Strategy
Source: Angew Chem Int Ed Engl. 2025 Jun 18;64(33):e202508078. doi: 10.1002/anie.202508078 (PMC12338415; doi:10.1002/anie.202508078)

**Supporting Information**

**Overcoming Photochemical Limitations in Covalent Organic Frameworks: Low-energy Light Driven Selective ^1^O_2_ Generation Achieved by Donor-Acceptor Strategy**

Jikuan Qiu,^[a]^ Hanping Zhai,^[a]^ Yuling Zhao,*^[a]^ Yucheng Jin,^[b]^ Zhiyong Li,^[a]^ Huiyong Wang,^[a]^ Zhongping Li,^[b]^ Jianji Wang*^[a]^ and Jong-Beom Baek*^[b]^

^[a]^School of Chemistry and Chemical Engineering, Key Laboratory of Green Chemical Media and Reactions, Ministry of Education Henan Normal University, 46 Jianshe Road E., Xinxiang, Henan 453007, P. R. China

E-mail: ylzhao@htu.edu.cn; jwang@htu.edu.cn

^[b]^Department of Energy and Chemical Engineering/Center for Dimension-Controllable Organic Frameworks, Ulsan National Institute of Science and Technology, 50 UNIST-gil, Eonyang-eup, Ulju-gun, Ulsan, 44919, Republic of Korea

E-mail: [jbbaek@unist.ac.kr](mailto:jbbaek@unist.ac.kr)

**Table of Contents**

1. Materials synthesis and characterization…….…………………………………... S3

2. Photocatalytic procedures…….…………………………………………………. S7

3. Computational details............................................................................................ S9

4. Figures S1-S35...................................................................................................... S10

5. Tables S1-S5………………………………...………………………...………... S45

6. References………………………………………………………………………. S51

7.^1^H NMR spectrum of the corresponding products.................................................S52

**Section S1. Materials synthesis and characterization**

***Materials***

A series of o-phenylenediamine and benzaldehyde derivatives such as o-phenylenediamine (99.5%), benzaldehyde (99%), p-tolualdehyde (99%), 4-nitrobenzaldehyde (98%), 4-nitrobenzal- dehyde (99%), 4-bromobenzaldehyde (99%), 4-hydroxybenzaldehyde (98%), 4-cyanobenzalde- hyde (98%), 2-thiophenecarboxaldehyde (98%), cyclohexanecarboxaldehyde (98%), indole-5- carboxaldehyde (97%) were purchased were from Alfa Aesar (Shanghai, China). The 1,3,5-tris (4-aminophenyl)benzene (98%), 1,3,5-benzenetricarboxaldehyde (98%) and various sulfides were purchased from Adamas Reagent (Shanghai, China). Acetic acid (99.7 %), anhydrous acetonitrile (CH_3_CN) (≥ 99 %, AR), tetrahydrofuran (THF) (≥ 99%, AR), anhydrous EtOH (≥ 99.5%), and acetone (≥ 99.5%) were purchased from commercially and used without further purification.

***Synthesis of* 2,7,12-Triamino-5H-diindeno[1,2-a:1',2'-c]fluorene-5,10,15-trione (*Tro)***

*Synthesis of* *trimerization of indane-1,3-dione (****2****)*

Typically, **2** was synthesized by following the previously reported procedures with slight modification ^[1]^. A 500 mL round-bottom flask was charged with 200 mL concentrated sulphuric acid, cooling with ice barth. Then, 1,3-indanedione **1** (20 g) was added portion-wise to H_2_SO_4_ under stirring. The reaction mixture was heated to 100 °C for 3 h. After the reaction finished, the mixture was poured onto ice (1 L). The participate was filtered and washed with acetone. The compound **2** was obtained by recrystallization from nitrobenzene as yellow solid (10.3 g).

*Synthesis of* *Nitration of 5H-tribenzo(a,f,k)trindene-5,10,15-trione (****3****)*

5.0 g of Trimerization of indane-1,3-dione (**2**) is suspended in 150 ml of concentrated sulfuric acid at -10°C. Subsequently, 0.036 mol of 100% nitric acid is added dropwise and the mixture is stirred for 1 h at -10°C. Then, the mixture was stirred 12 hours at room temperature. The resulting compound **3** is then poured onto ice (1 L), extracted with EtOEt, washed with water. After drying, the product is recrystallized from nitrobenzene with 85% yield.

*Synthesis of* *Tro (****4****)*

A mixture of **3** (5.0 g) of and activated Raney nickel is suspended in DMF (100ml) at 120°C. Then, hydrazine hydrate (98%, 25 mL) is added slowly under N_2_ atmosphere, and the mixture is re-fluxed for 2 h. The solvent was removed by distillation, and the crude product is extracted with EtOEt and purified by column chromatography to obtain the **4**.


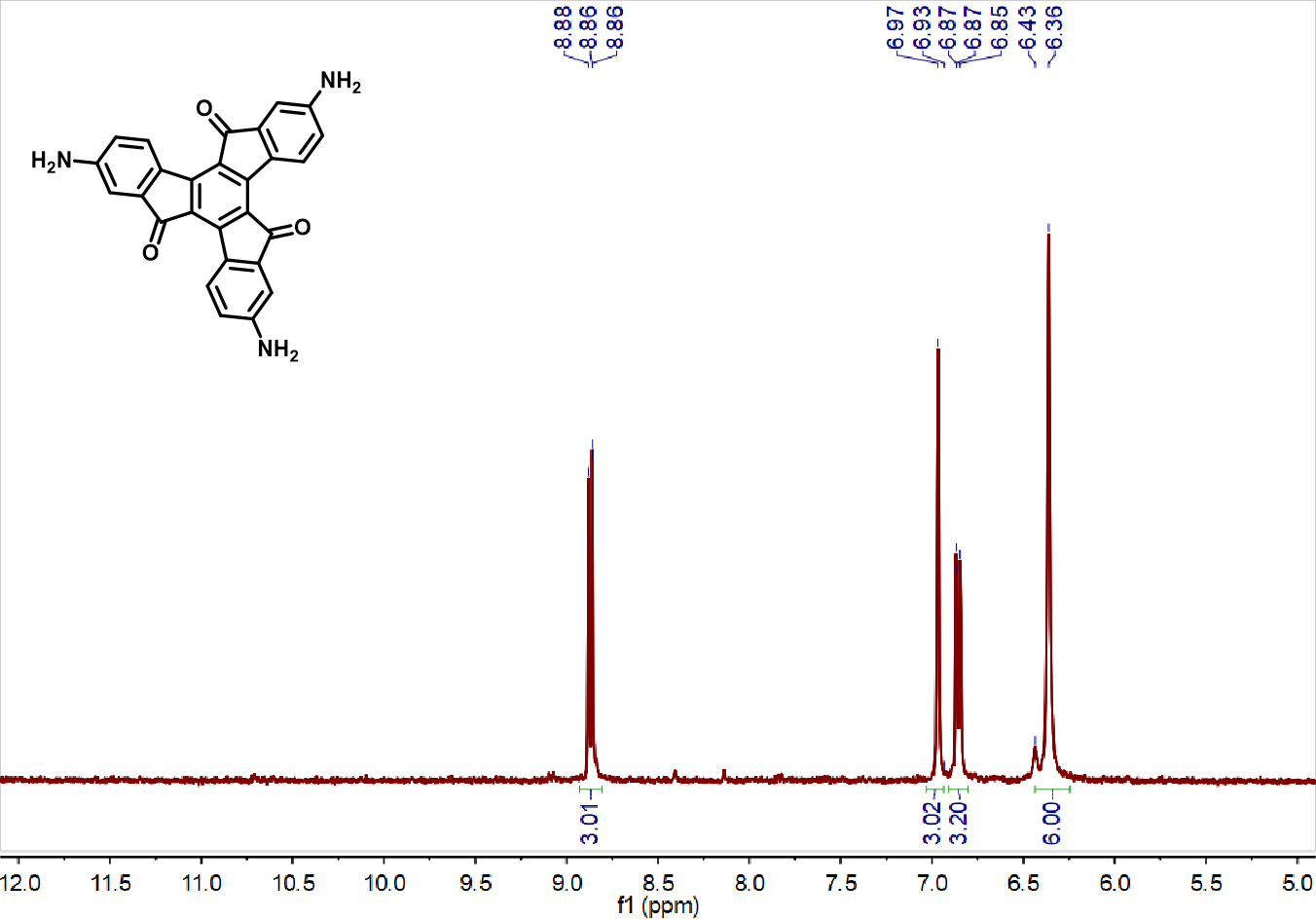


***Synthesis of TpbTfb-COF***

Typically, 1,3,5-Tris(4-aminophenyl)benzene (Tpb, 0.075 mmol), 1,3,5-Benzenetricarbox- aldehyde (Tfb, 0.075 mmol), dioxane (1.5 mL), and 1,3,5-trimethylbenzene (1.5 mL) were charged into a glass tube. After dispersing evenly, 6 M acetic acid (0.15 mL) was added, followed by the degassing procedure using freeze-pump-thaw cycles three times. The glass vial was then flame-sealed and the mixture was allowed to react at 120 °C for 3 days. After collecting by filtration, the precipitate was washed with ethanol, tetrahydrofuran, and acetone. Finally, the solid was dried under vacuum at 60 °C for 12 h to obtain TpbTfb-COF as orange powder.

***Synthesis of TroTfb-COF***

Typically, 2,7,12-Triamino-5H-diindeno[1,2-a:1',2'-c]fluorene-5,10,15-trione (Tro, 0.075 mmol), 1,3,5-Benzenetricarboxaldehyde (Tfb, 0.075 mmol), N-Methylpyrrolidone (1.5 mL), and N,N-Dimethylacetamide (1.5 mL) were charged into a glass tube. After dispersing evenly, 6 M acetic acid (0.3 mL) was added, followed by the degassing procedure using freeze-pump-thaw cycles three times. The glass vial was then flame-sealed, and the mixture was allowed to react at 120 °C for 3 days. After collecting by filtration, the precipitate was washed with ethanol, tetrahydrofuran, and acetone. Finally, the solid was dried under vacuum at 60 °C for 12 h to obtain TroTfb-COF as brick-red powder.

***Characterization***

Power X-ray diffraction (PXRD) data were obtained with an X’ Pert3 powder diffractometer at 40 kV and 40 mA with Cu Kradiation from 2*θ* = 2° to 40° in 0.05° increment. Fourier transform infrared (FT-IR) spectra of the samples were collected on a Spectrum 400 spectrometer (Perkin-Elmer). All samples were ground into a powder with KBr and pelletized before the measurements. Nitrogen adsorption and desorption isotherms were measured at 77 K using an ASiQwin Quantachrome instrument. The samples were treated at 120 °C for 8 h before measurements. Specific surface areas were calculated from the adsorption data using Brunauer-Emmett-Teller (BET) methods. Pore size distribution data were calculated based on the nonlocal density functional theory (NLDFT) model. Field emission scanning electron microscopy (FE-SEM) observations were performed on a Hitachi SU8010 microscope operated at an accelerating voltage of 10.0 kV. ^1^H nuclear magnetic resonance (^1^H NMR) spectra were recorded by a Bruker Advance III 400 MHz NMR spectrometer with chemical shift in ppm. UV-vis spectra were collected on a Shimadzu Corporation UV-2600 220V CH spectrometer. Photoluminescence and Time-resolved PL decay spectra were collected on a Horiba FluoroLog-3 in air atmosphere. ESR spectra were recorded on an EMXplus.

***Electrochemical Characterization***

Photoelectrochemical and electrochemical measurements including photocurrent test, electro- chemical impedance spectra (EIS), and Mott-Schottky plot were performed with an electro- chemical working station (CHI 760E) via a three-electrode quartz cell. Ag/AgCl electrode and Pt mesh were immersed in aqueous sodium sulfate solution (0.5 M) to serve as the reference electrode and the counter electrode, respectively. The working electrode was prepared as follows: COF material (2 mg) was dispersed in a mixed solution of ethanol (0.2 mL) and Nafion (5 μL) to form a homogeneous slurry. Subsequently, 200 μL of the slurry was transferred and coated on an ITO glass plate (0.5 cm × 0.5 cm), and then dried at 60 °C. For photocurrent measurements, irradiation conditions were the same as photocatalytic tests. In the EIS measurements, the frequency limit was set in the range from 100 kHz to 0.01 Hz with 5 mV of voltage amplitude at the open-circuit potential. For Mott-Schottky plot measurements, the samples were tested with different frequencies at 500, 1000, and 1500 Hz.

***Electron spin resonance (ESR) test***

The ESR measurements were carried out on an EMXplus, functioning at the frequency of X-band (8.7-9.6 GHz). Typically, 50 µL of aqueous suspension of COFs (5 g/L) was mixed with 500 µL of 2,2,6,6-tetramethylpiperidine (TEMP, 50 mM) solution. The O_2_•^-^ trapping ESR test was conducted in absolute methanol, with the same protocol above, except for using 5,5-dimethyl-1-pyrroline-N-oxide (DMPO) as the spin-trapping agent.

**Section S2. Photocatalytic procedures**

***General procedure for photocatalytic Oxidation of Sulfides***

A 10 mL Schlenk tube equipped with a stir bar, was loaded with the mixture of sulfide (0.05 mmol), TroTfb-COF (10 mg) and CH_3_CN (5 mL). The reaction mixture underwent irradiation in a photo-reactor equipped with 300 W Xenon lamp emitting light at 630 nm, along with a cooling fan, for duration of 5-10 hours under an O_2_ atmosphere. The light intensity was controlled at 0.355 W cm^-2^ determined by an International light technology 950 spectroradiometer. Upon completion of the reaction, the photocatalyst was collected by centrifugation, and the solvent was concentrated under reduced pressure. The resulting residue was subjected to purification via flash chromatography on silica gel using a petroleum ether/ethyl acetate mixture as the eluent, leading to the isolation of the desired product. Quenching experiments have been carried out to identify ROSs, in which isopropanol (iPrOH), DBACO, *p*-benzoquinone (BQ) serve as the scavenger for •OH, ^1^O_2_, and O_2_^•-^ radical, respectively.^[2]^

***General procedure for photocatalytic synthesis of benzimidazole***

A 10 mL Schlenk tube equipped with a stir bar, was loaded with the mixture of o-phenylenediamine (0.25 mmol), aldehyde (0.25 mmol), TroTfb-COF (3 mg) and C_2_H_5_OH (3 mL). The reaction mixture underwent irradiation in a photo-reactor equipped with 20 W red LEDs emitting light at 630 nm, along with a cooling fan, for duration of 3 hours under an O_2_ atmosphere. Upon completion of the reaction, the photocatalyst was collected by centrifugation, and the solvent was concentrated under reduced pressure. The resulting residue was subjected to purification via flash chromatography on silica gel using a petroleum ether/ethyl acetate mixture as the eluent, leading to the isolation of the desired product.

***Recyclability of catalysts***

For the recyclability test, the catalysts were separated from the reaction mixtures by centrifugation and washed with THF three times. After dried at 80 °C in an oven, the catalysts were reused for the next run under the optimum reaction conditions.

***Photocataltic degradation of 4-CP***

Typically, catalyst (15 mg) is added into the aqueous solution of 4-CP (50 mL, 10 mg L^−1^) in a quartz reactor. Subsequently, the suspension was completely agitated for 15 min to establish an adsorption desorption equilibrium with 300 W Xenon lamp emitting light at 630 nm. The light intensity was controlled at 0.355 W cm^-2^ determined by an International light technology 950 spectroradiometer. Then, at specific time intervals, 1 mL water samples were taken out from the quartz reactor using a syringe and filtered through a 0.22 µm polytetrafluoroethylene (PTFE) filter. The reproducibility of the process was confirmed by performing each of the experiments at least three times. The concentration of 4-CP was analyzed by HPLC and the mobile phases and detection wavelengths were set as methanol/water (0.1% methanoic acid) (70:30, v/v) with λ= 225 nm. The intermediates were analyzed by HPLC and GC-MS. The percentage of degradation efficiency is defined by the following equation^[3]^:

Degradation efficiency (%) = *C*_t_/*C*_0_

where *C*_0_ is the initial pollutant concentration, *C*_t_ is the concentration at a certain time *t* during the degradation process.

**Section S3. Computational details**

The models for each of the possible structures were constructed and compared their simulated PXRD patterns with the experimental data. The structural models were geometrically optimized in the Vienna ab initio simulation package (VASP) code^[4]^, which is based on the projector-augmented wave (PAW) method^[5]^. To describe the exchange correlation interactions, a generalized gradient approximation (GGA)^[6]^ with the Perdew-Burke-Ernzerhof (PBE)^[7]^ functional was employed. The plane-wave cutoff energy was set to 400 eV. For geometry optimizations, the Brillouin zone was sampled with the Gamma centered Monkhorst-Pack^[8]^ scheme K-point grid of 1×1×1. All the structures were optimized using the conjugant gradient method with an energy convergence criterion of 1×10^−5^ eV and a force convergence criterion of 0.02 eV/Å. Following geometry optimization, Pawley refinement was carried out using Reflex software package. The Pawley refinement was performed to optimize the lattice parameters iteratively until the Rwp value converges and the overlay of the observed with refined profiles shows good agreement.

Stacking energies are also calculated as E(bilayer)–[E(top) + E(bottom)]. Energies of each layer and the bilayer (top and side views) for TpbTfb-COF and TroTfb-COF were calculated using the VASP program. Along the z-direction, a large vacuum space of 50 Å was added to avoid interactions between the nearest-neighbor unit cells.

The structures of TpbTfb-COF and TroTfb-COF monomers were also optimized by using Gaussian 09 package^[9]^ at the B3LYP level.^[10,11]^ The electron-hole were calculated by B3LYP level exchange-correlation functional with the 6-31G(d) basis set. Frequency calculations were performed to ensure that the stability configuration has no imaginary frequency.^[12]^ The electron-hole and the overlaps distribution were calculated with Multiwfn 3.8.^[13]^

**Section S4. Figures S1-S35**


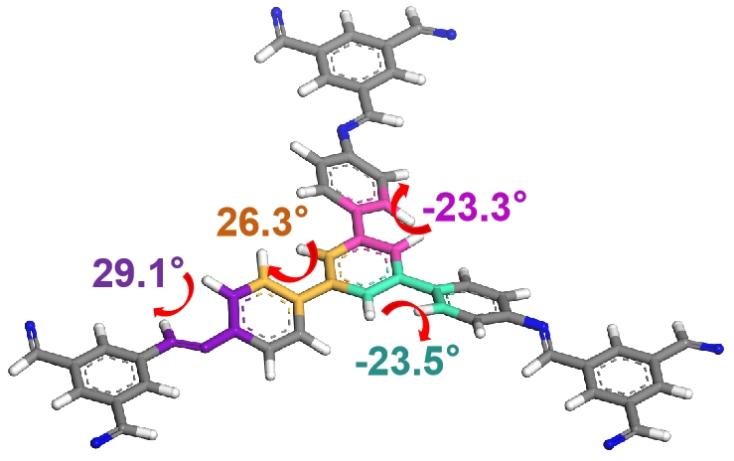


**Figure S1** Unit cell structure of TpbTfb-COF.


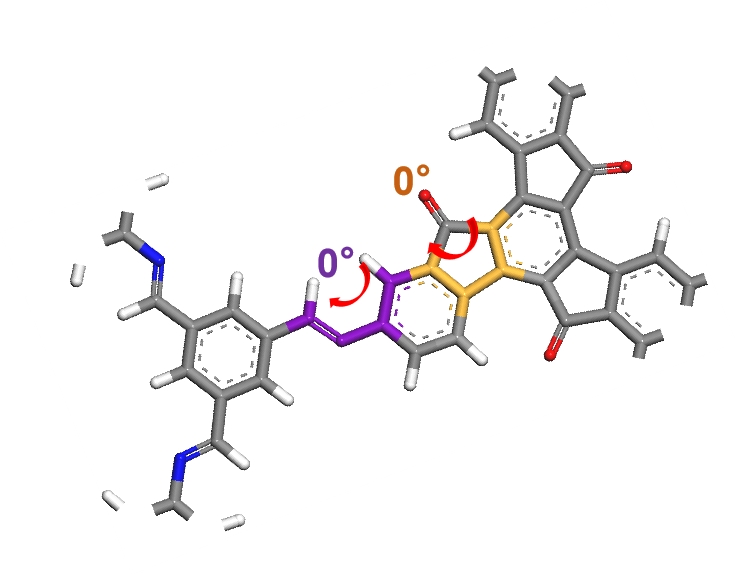


**Figure S2** Unit cell structure of TroTfb-COF.


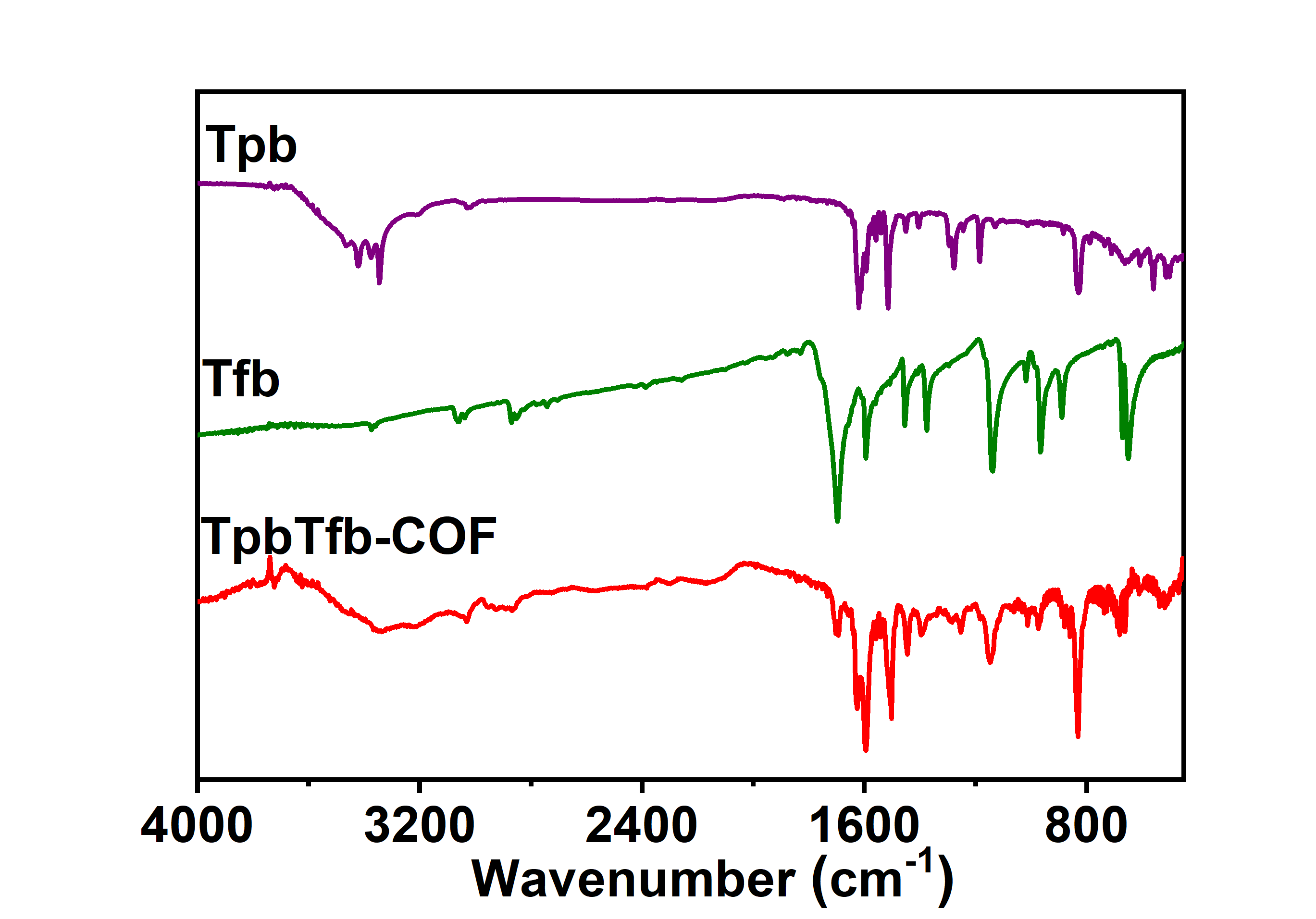


**Figure S3** FT-IR spectra of TpbTfb-COF and its corresponding monomers.


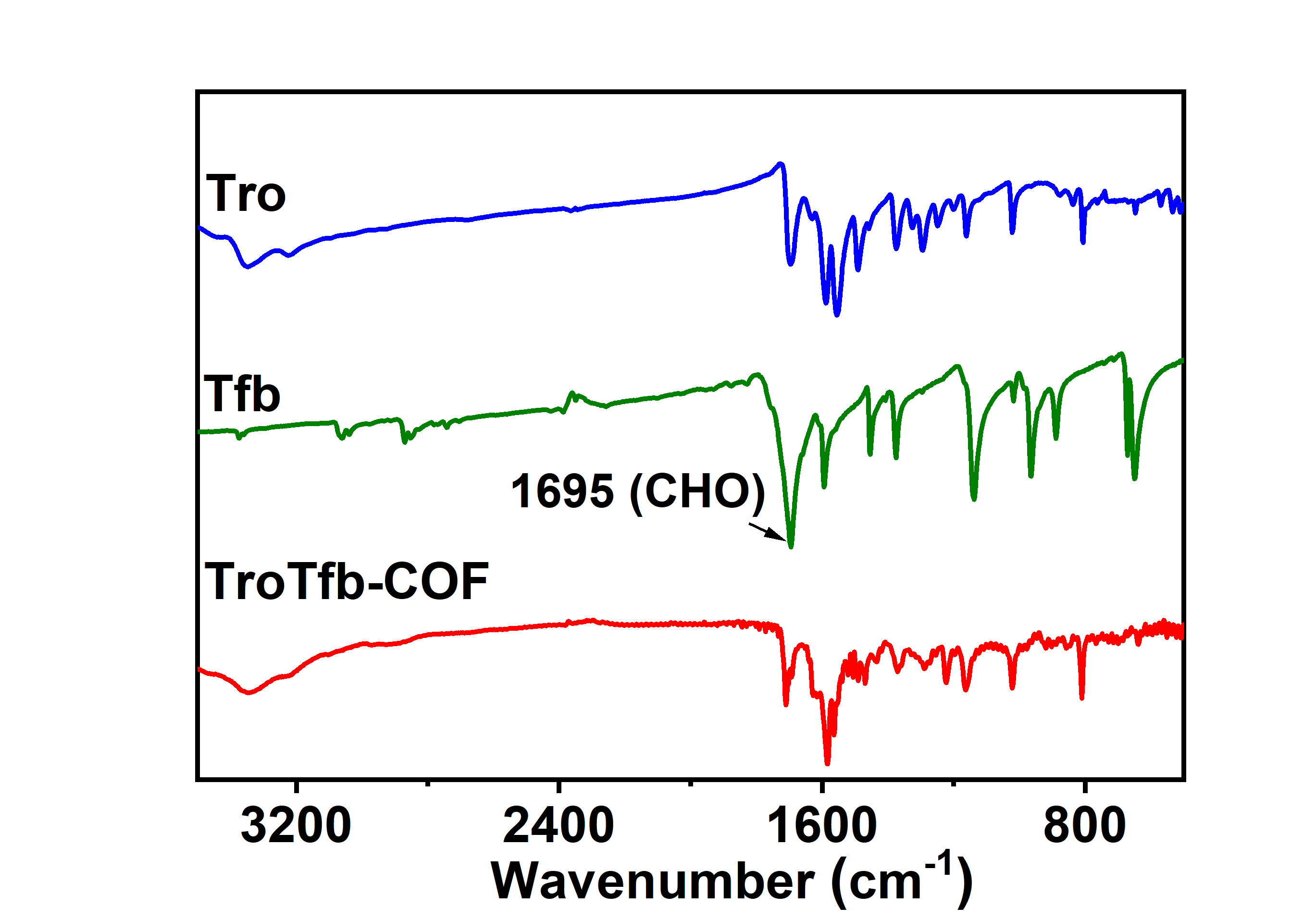


**Figure S4** FT-IR spectra of TroTfb-COF and its corresponding monomers.


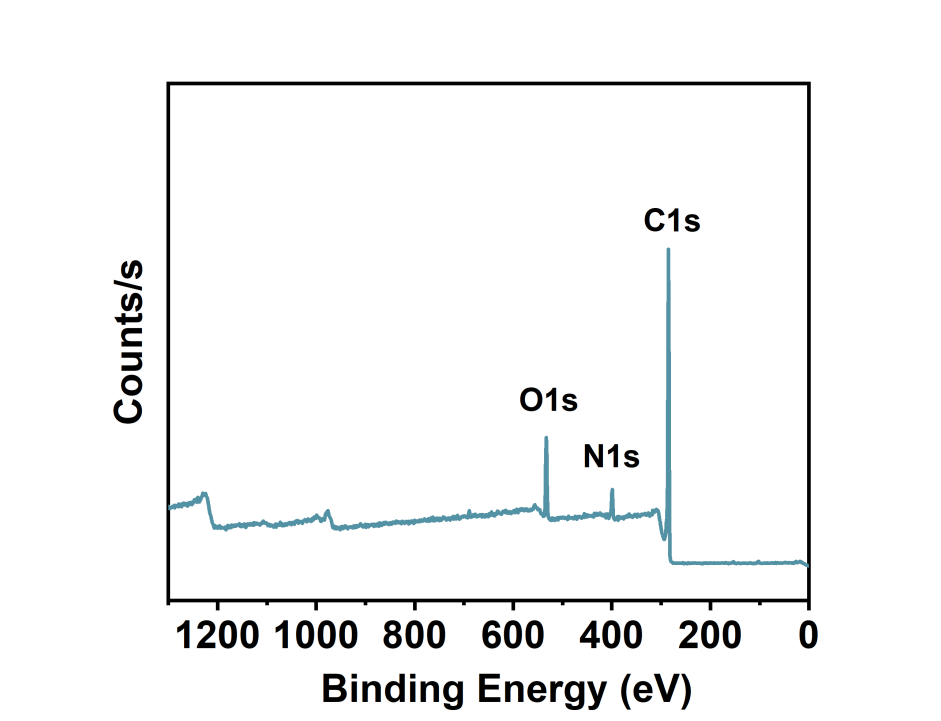


**Figure S5** High-resolution XPS spectrum of TpbTfb-COF.


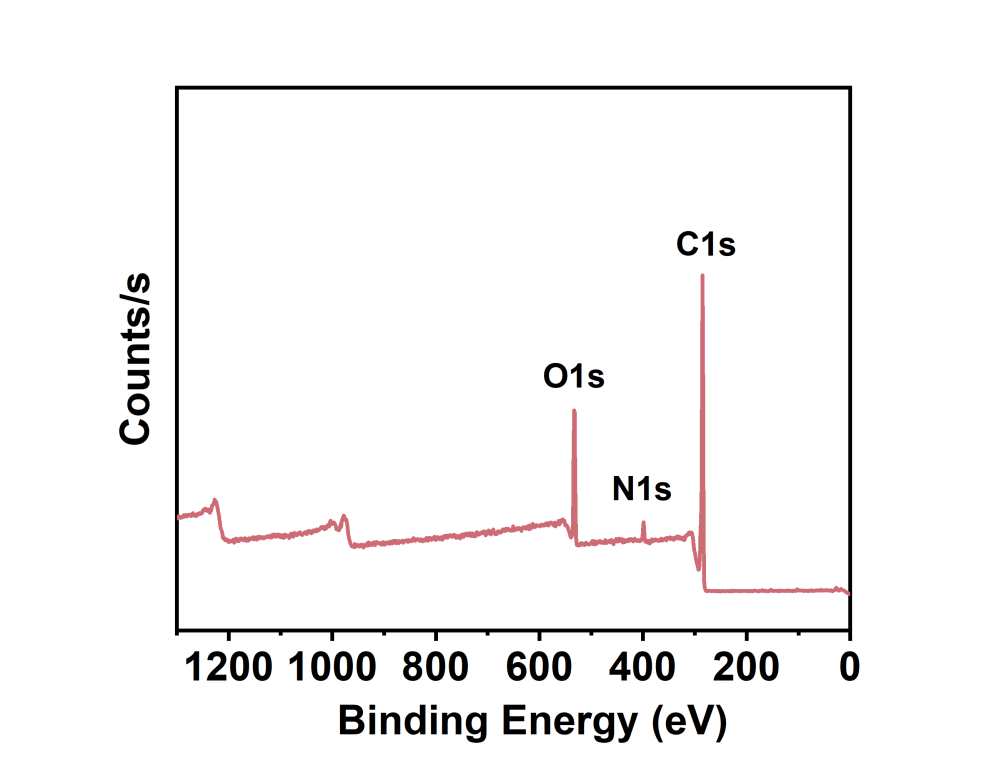


**Figure S6** High-resolution XPS spectrum of N 1s for TroTfb-COF.


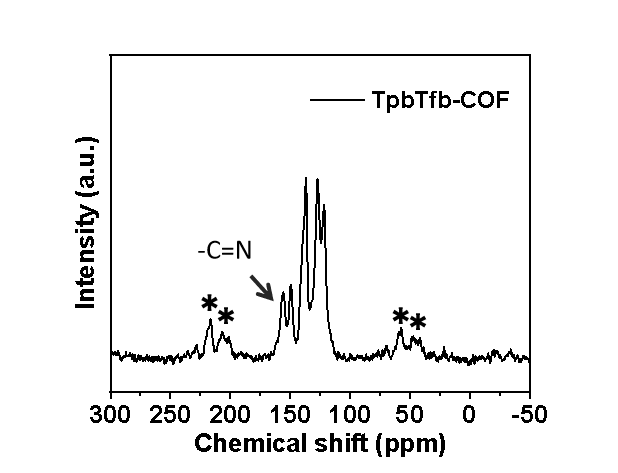


**Figure S7** ^13^C CP-MAS NMR spectra and carbon signal assignment of TpbTfb-COF. The asterisks denote the spinning side bands. The typical signal at ~155 ppm in the NMR spectrum confirmed the formation of C=N carbon.


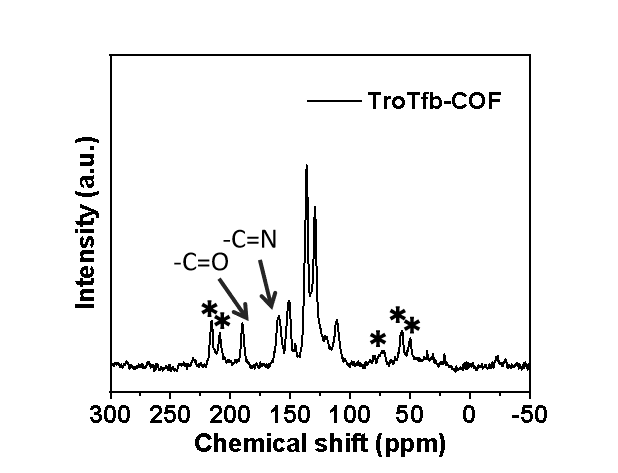


**Figure S8** ^13^C CP-MAS NMR spectra and carbon signal assignment of TroTfb-COF. The asterisks denote the spinning side bands. The typical signal at ~155 ppm in the NMR spectrum confirmed the formation of C=N carbon. The typical signal at ~185 ppm in the NMR spectrum confirmed the formation of C=O carbon.

**Figure S9** SEM images of TpbTfb-COF.

**Figure S10** SEM images of TroTfb-COF.


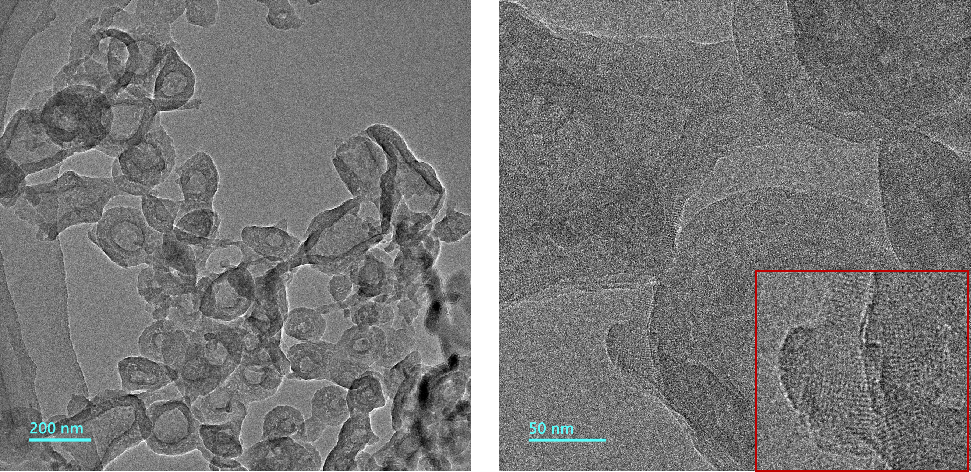


Figure S11 HRTEM images of TpbTfb-COF.


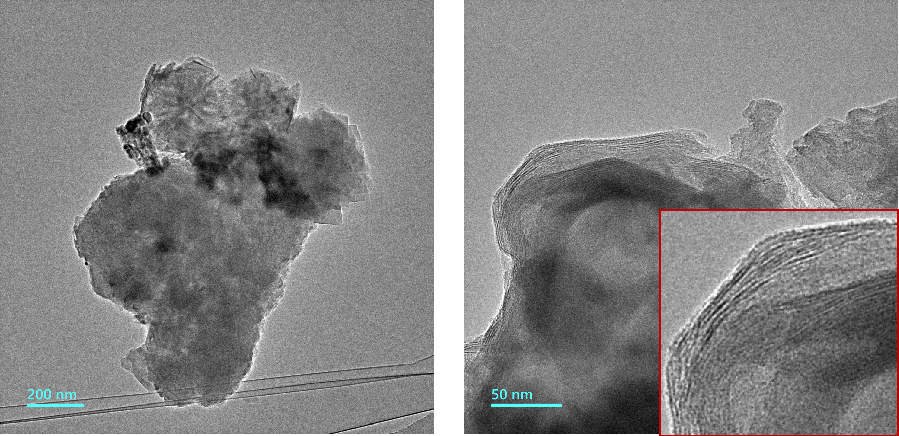


Figure S12 HRTEM images of TroTfb-COF.

**Figure S13** Experimental and simulated PXRD patterns (AB stacking) of TpbTfb-COF.

**Figure S14** Experimental and simulated PXRD patterns (AB stacking) of TroTfb-COF.

**Figure S15** PXRD patterns of TroTfb-COF treated with different solvents.


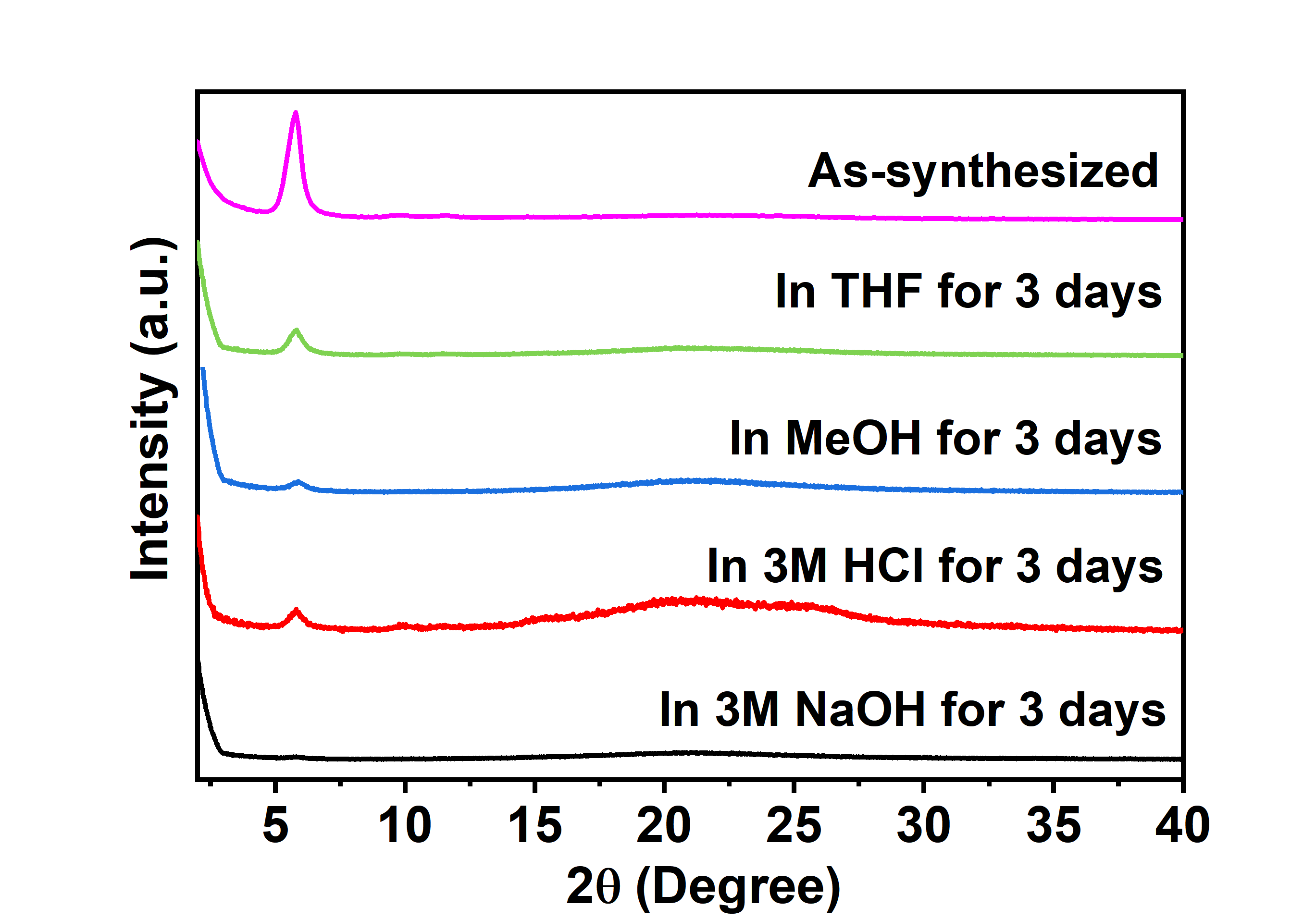


Figure S16 PXRD patterns of TroTfb-COF treated with different solvents.


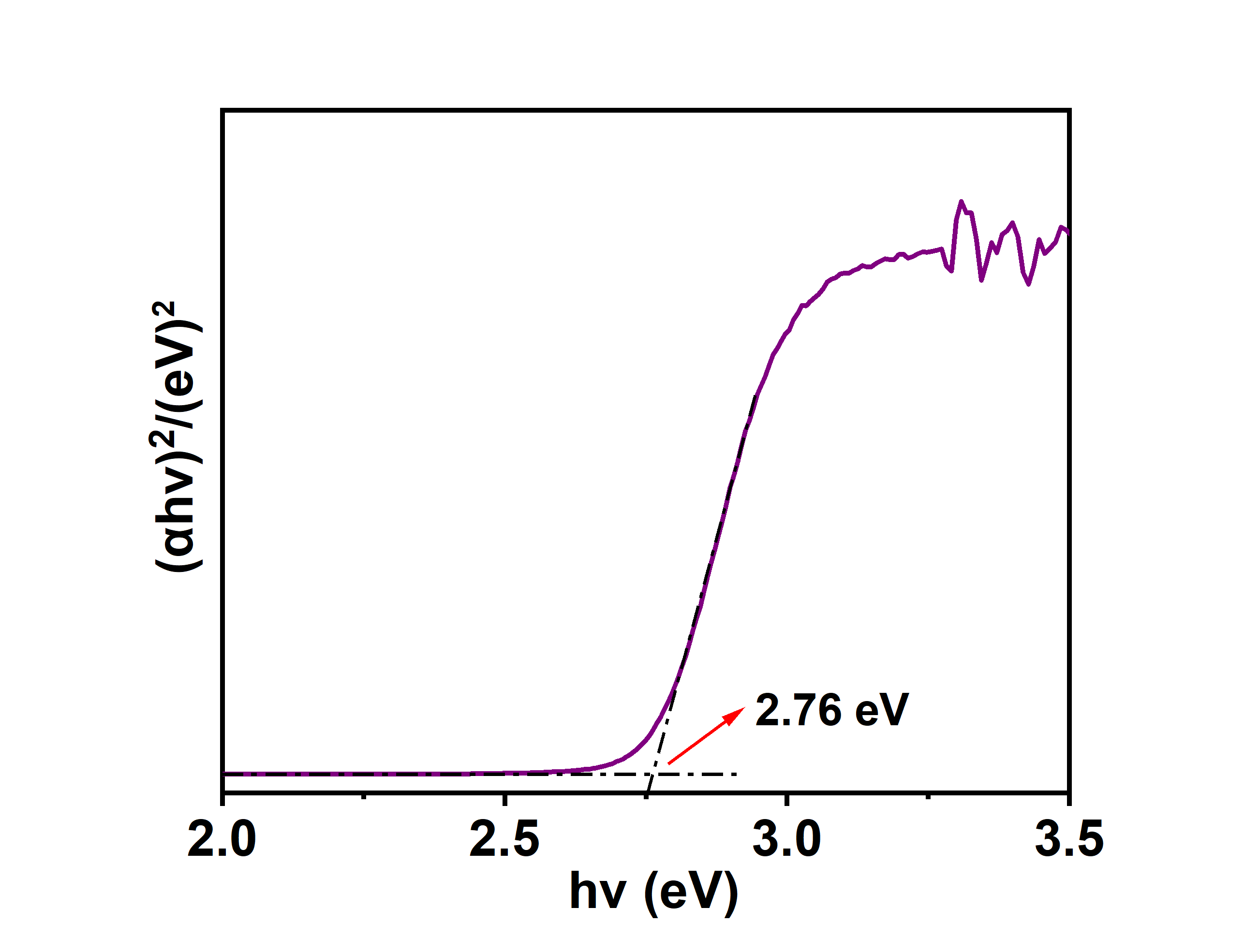


**Figure S17** Tauc plots with the band gaps of TpbTfb-COF.


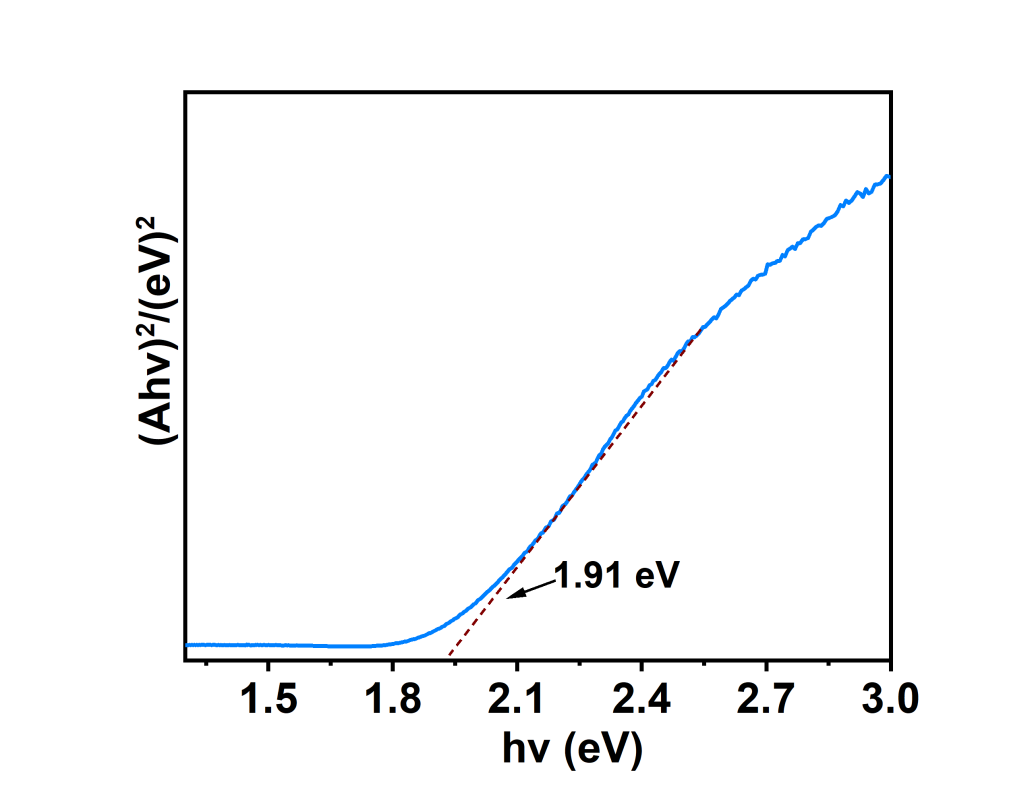


**Figure S18** Tauc plots with the band gaps of TroTfb-COF.


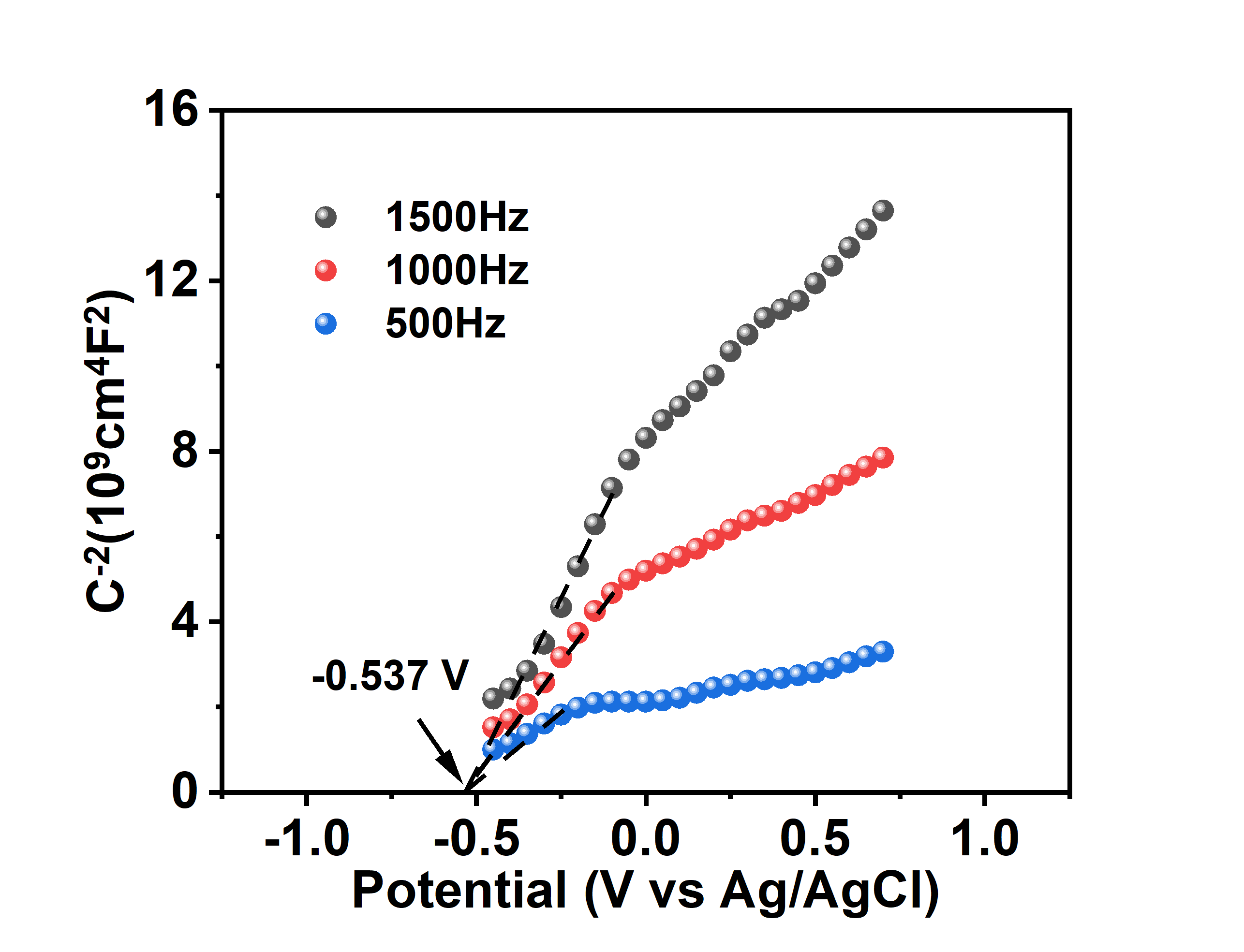


**Figure S19** Mott−Schottky plots of TpbTfb-COF in 0.1 M Na_2_SO_4_ aqueous solution at different frequencies.


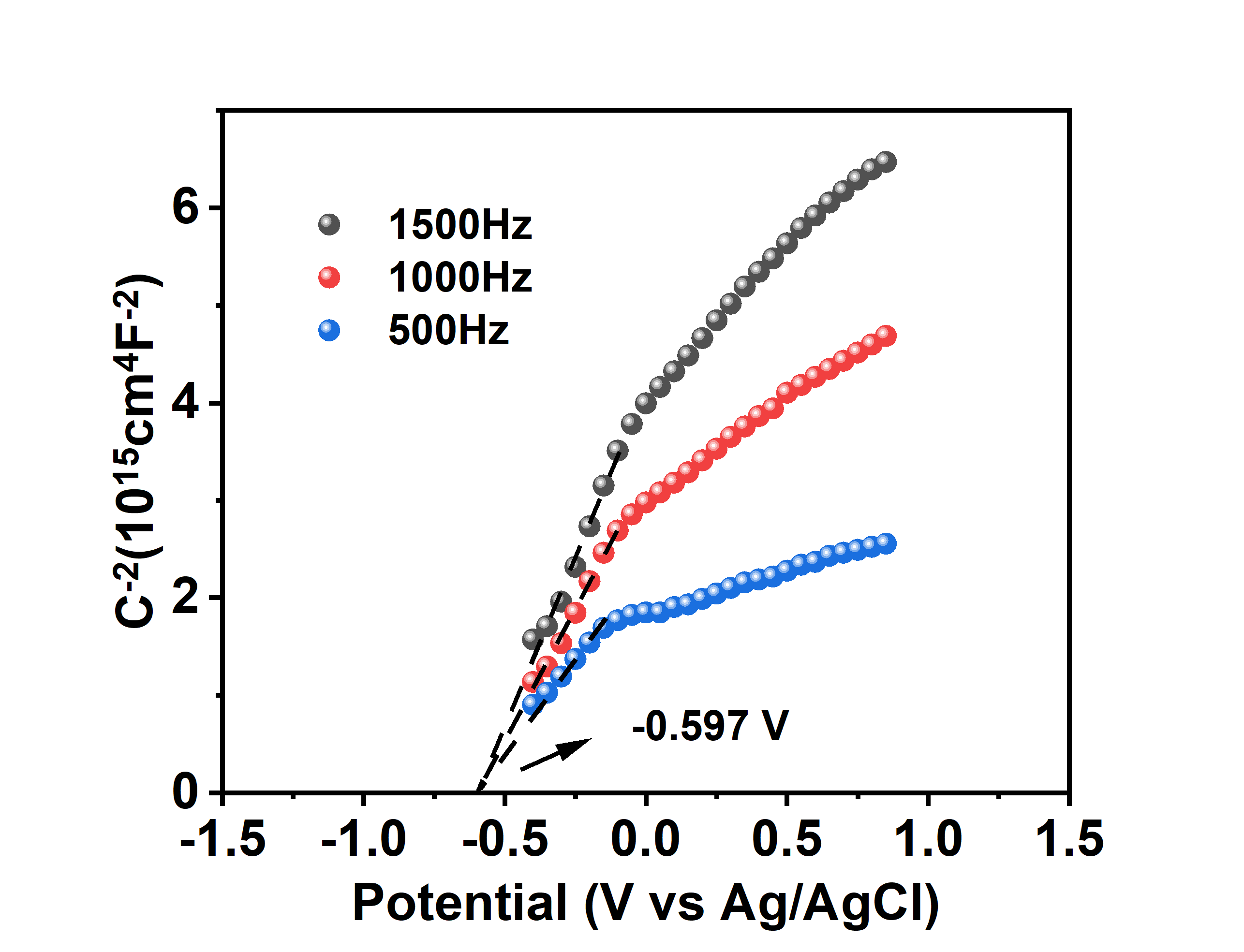


**Figure S20** Mott−Schottky plots of TroTfb-COF in 0.1 M Na_2_SO_4_ aqueous solution at different frequencies.


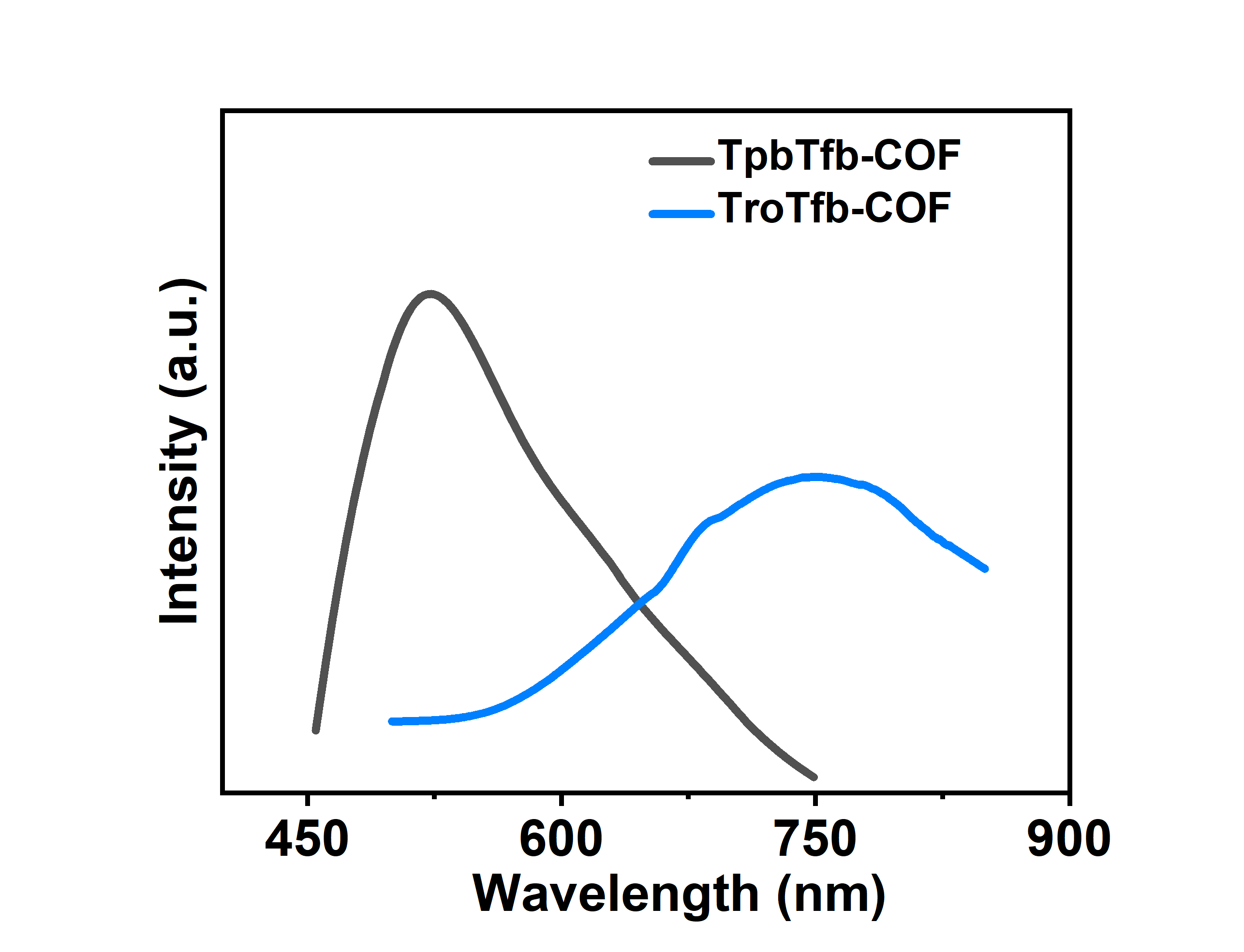


**Figure S21** Steady-state photoluminescence (PL) spectroscopy of TpbTfb-COF and TroTfb-COF.


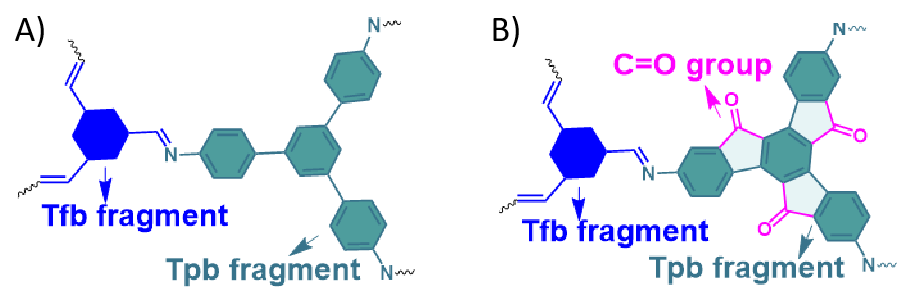


**Figure S22** Molecular structure decomposition of (A) TpbTfb-COF and (B) TroTfb-COF into Tpb fragment, Tfb fragment, and C=O group.


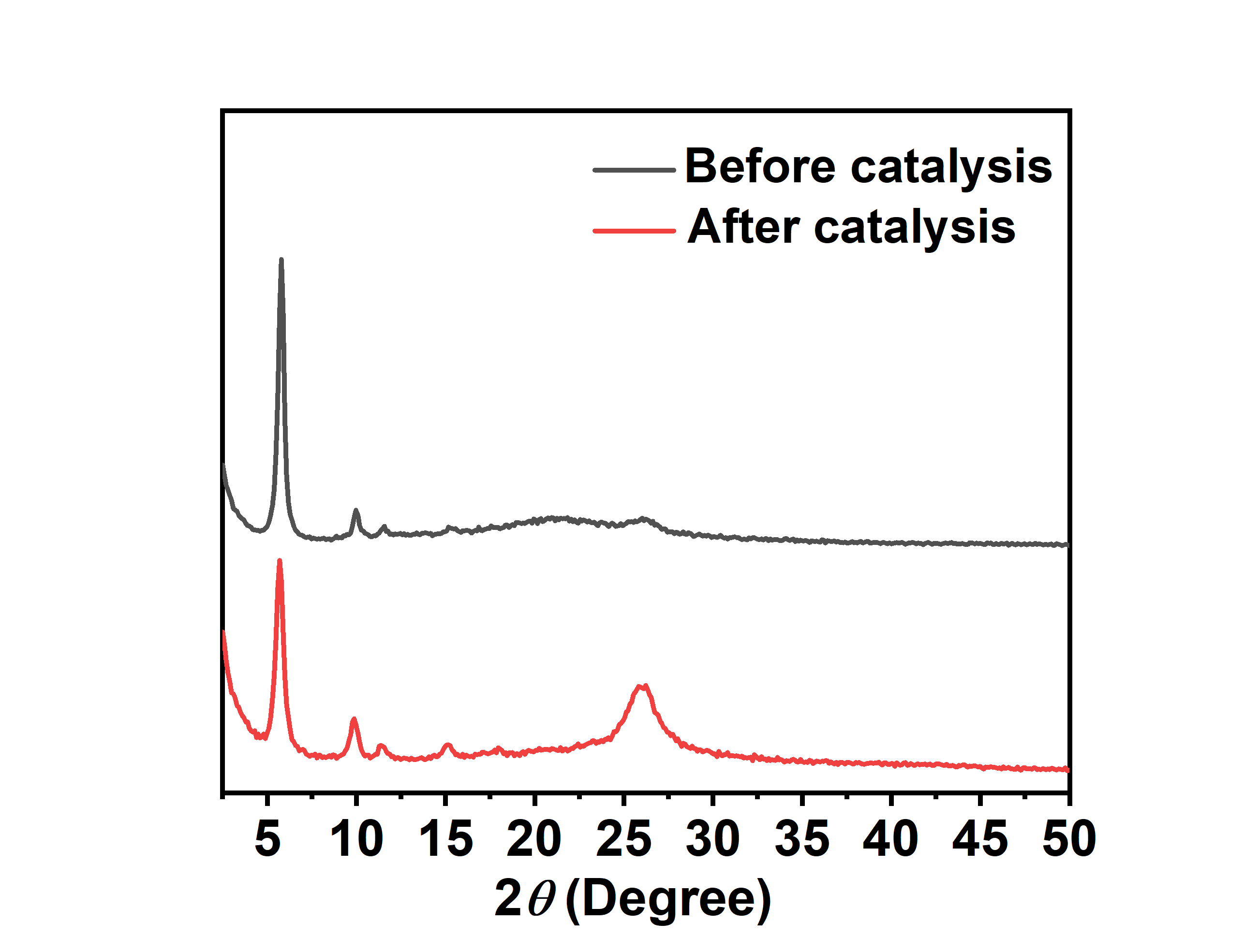


**Figure S23** PXRD-patterns of TroTfb-COF before and after the 10th cycle.


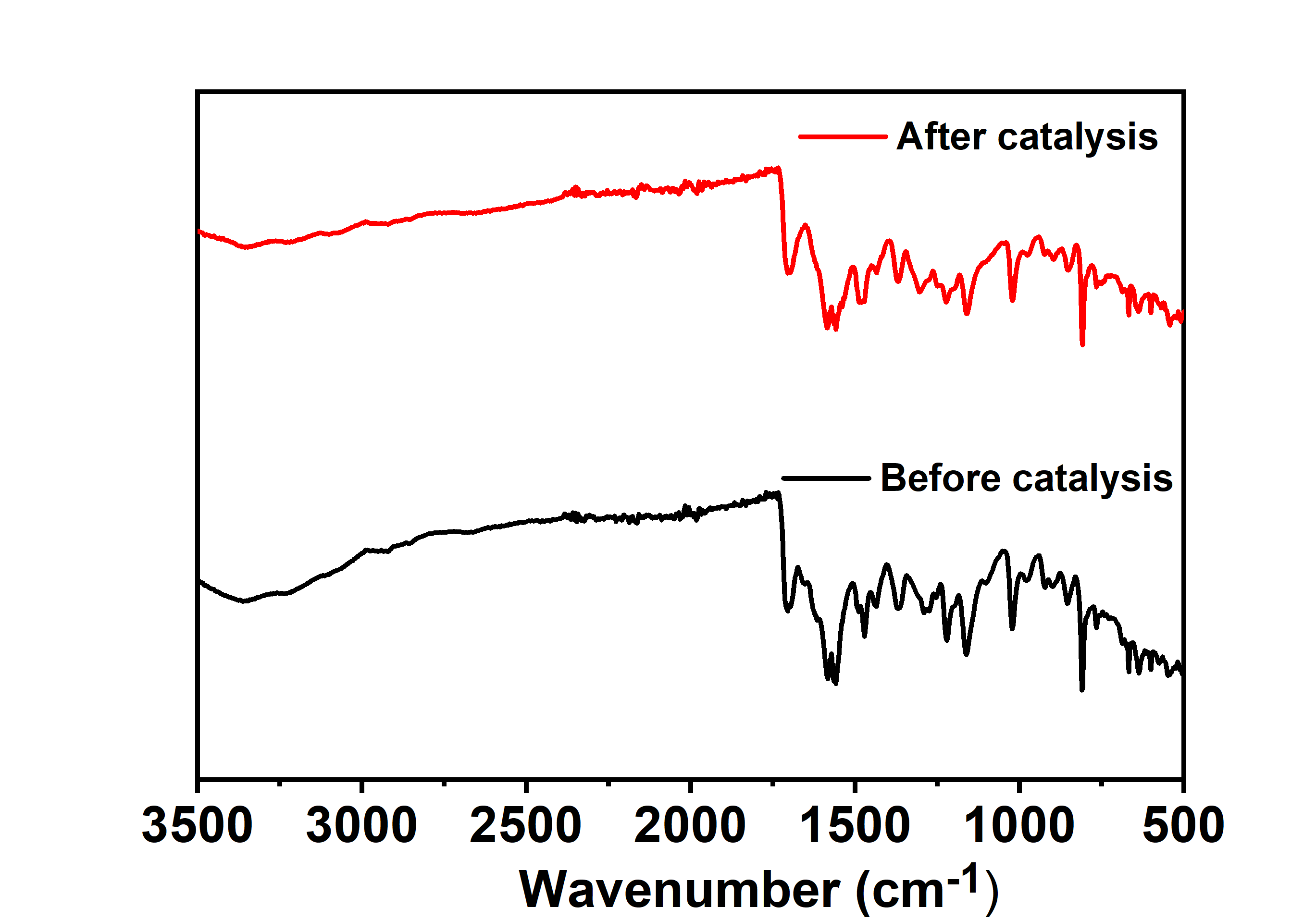


**Figure S24** IR patterns of TroTfb-COF before and after the 10th cycle.


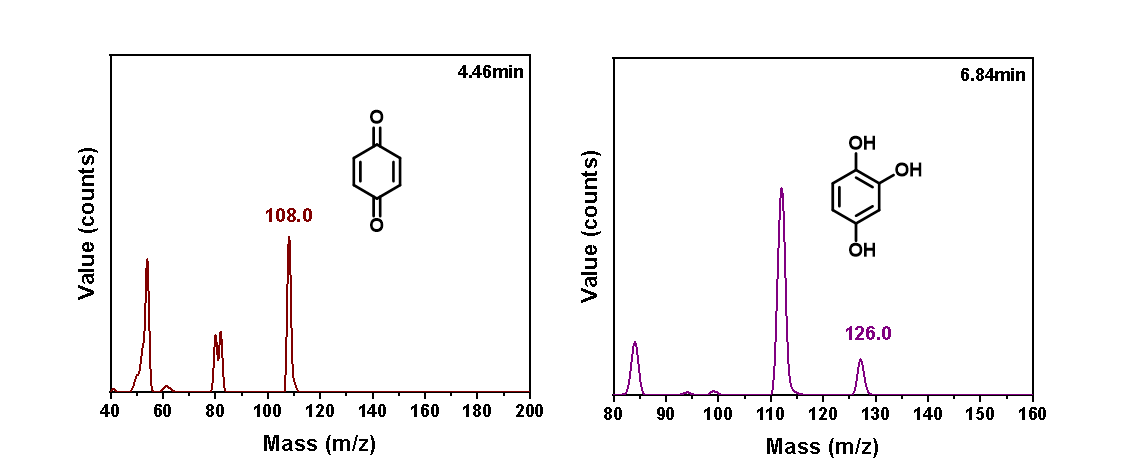


**Figure S25** Intermediate products of 4-CP degradation.


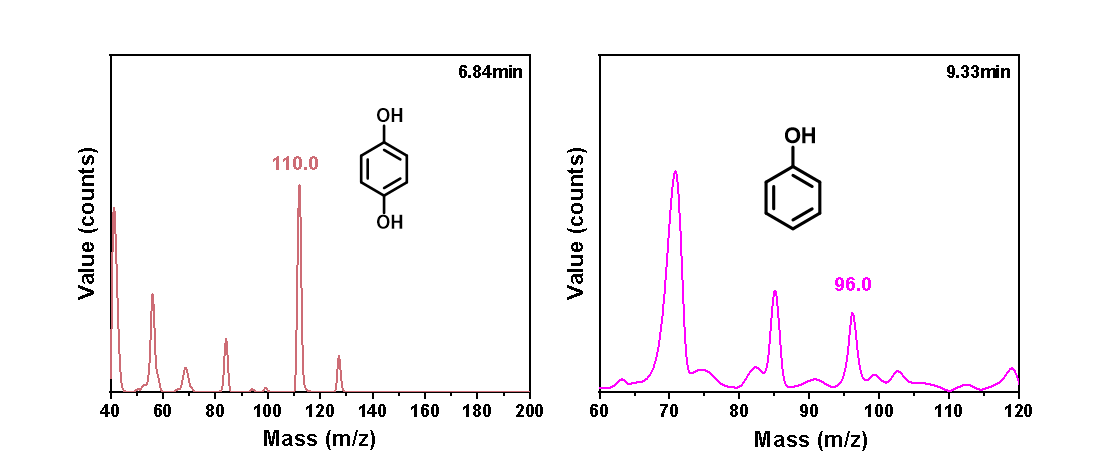


**Figure S26** Intermediate products of 4-CP degradation.


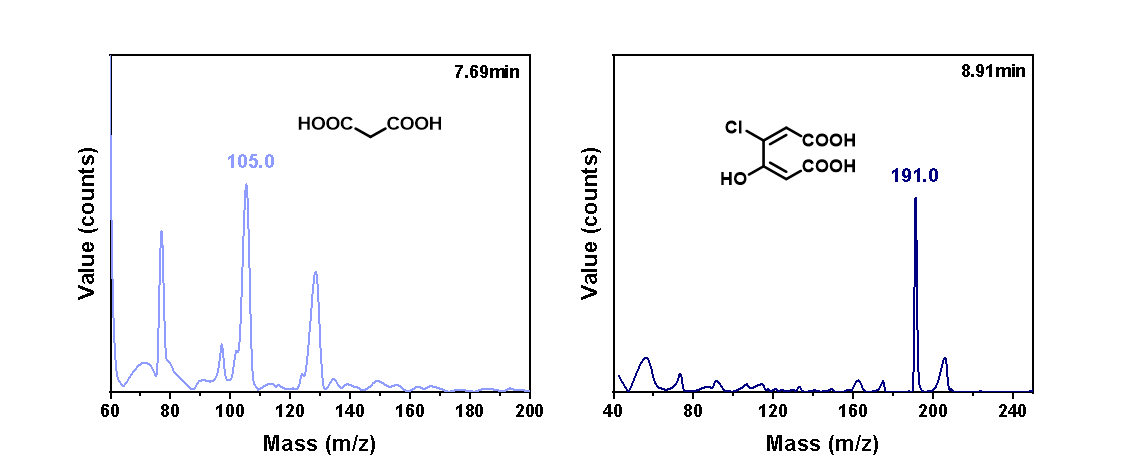


**Figure S27** Intermediate products of 4-CP degradation.


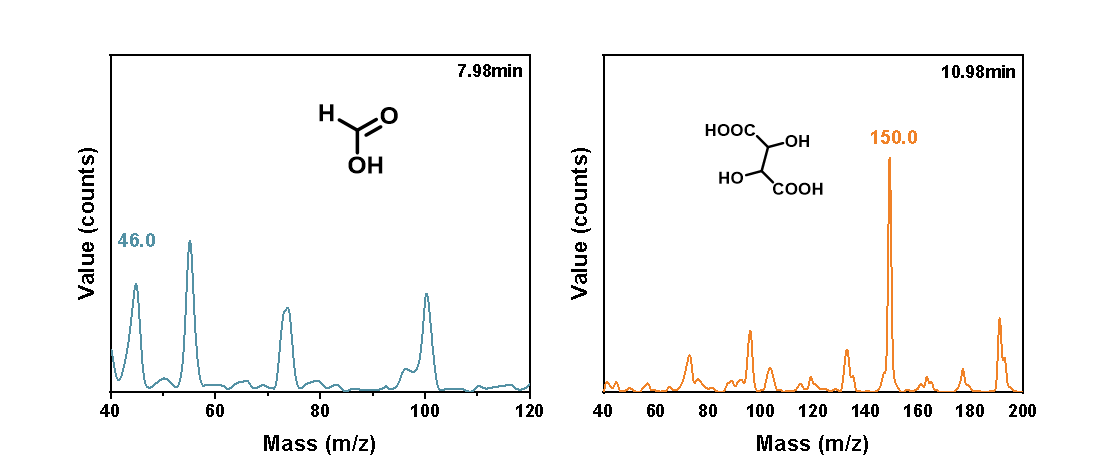


**Figure S28** Intermediate products of 4-CP degradation.


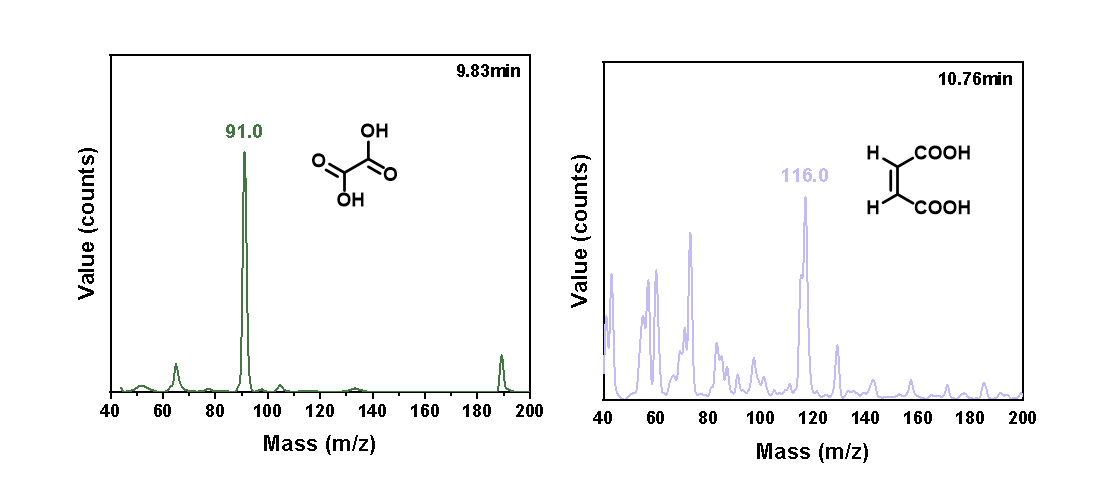


**Figure S29** Intermediate products of 4-CP degradation.


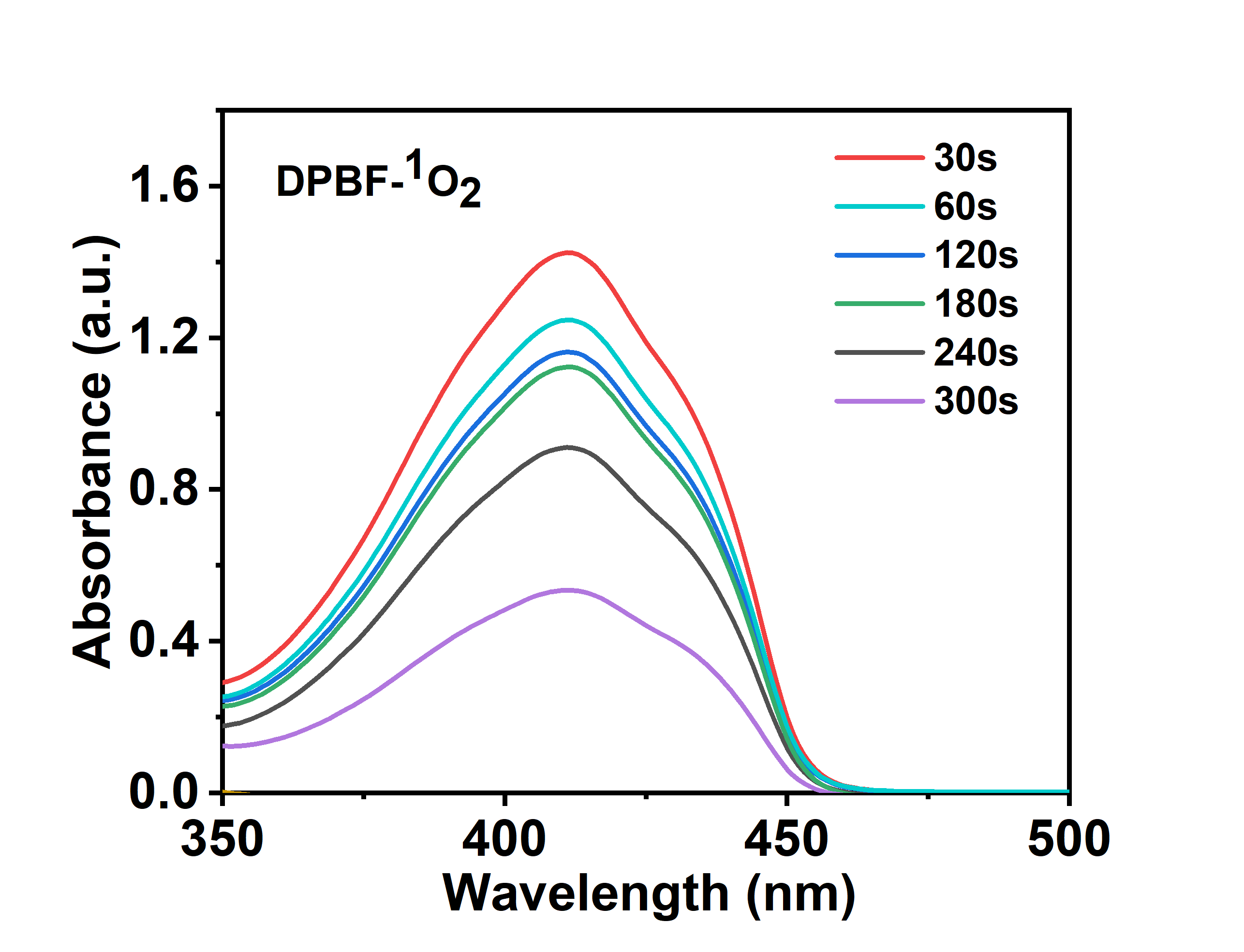


**Figure S30** UV-Vis absorption changes of DPBF at 410 nm with TroTfb-COF. Reaction conditions: [DPBF] = 1mM, [Cat.] = 0.25 g L^−1^ in 10 mL ethanol, O_2_ (1 atm), 25 °C, λ = 630 nm.


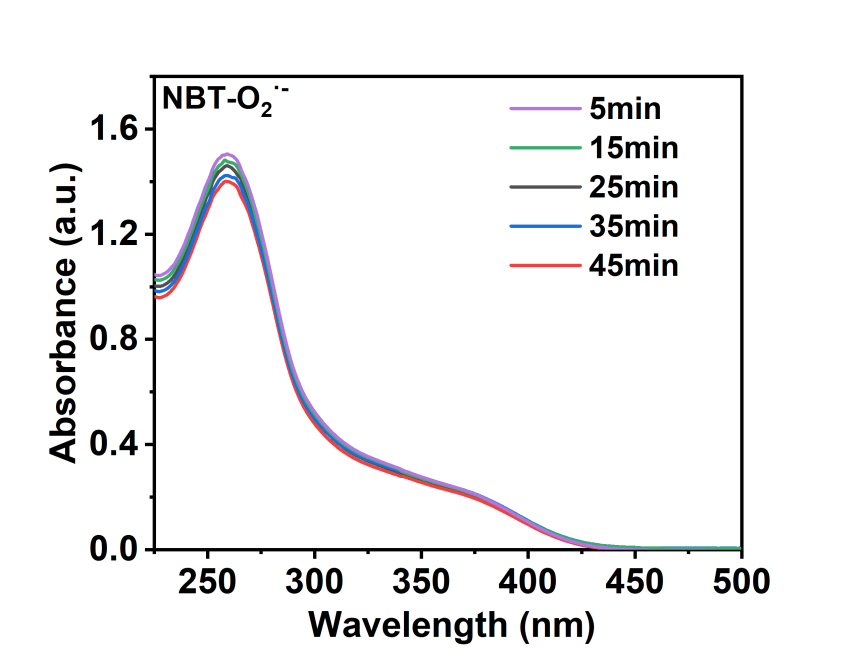


**Figure S31** UV-Vis absorption changes of NBT at 260 nm with TroTfb-COF. Reaction conditions: [NBT] = 1mM, [Cat.] = 0.25 g L^−1^ in 10 mL water, O_2_ (1 atm), 25 °C, λ = 630 nm.


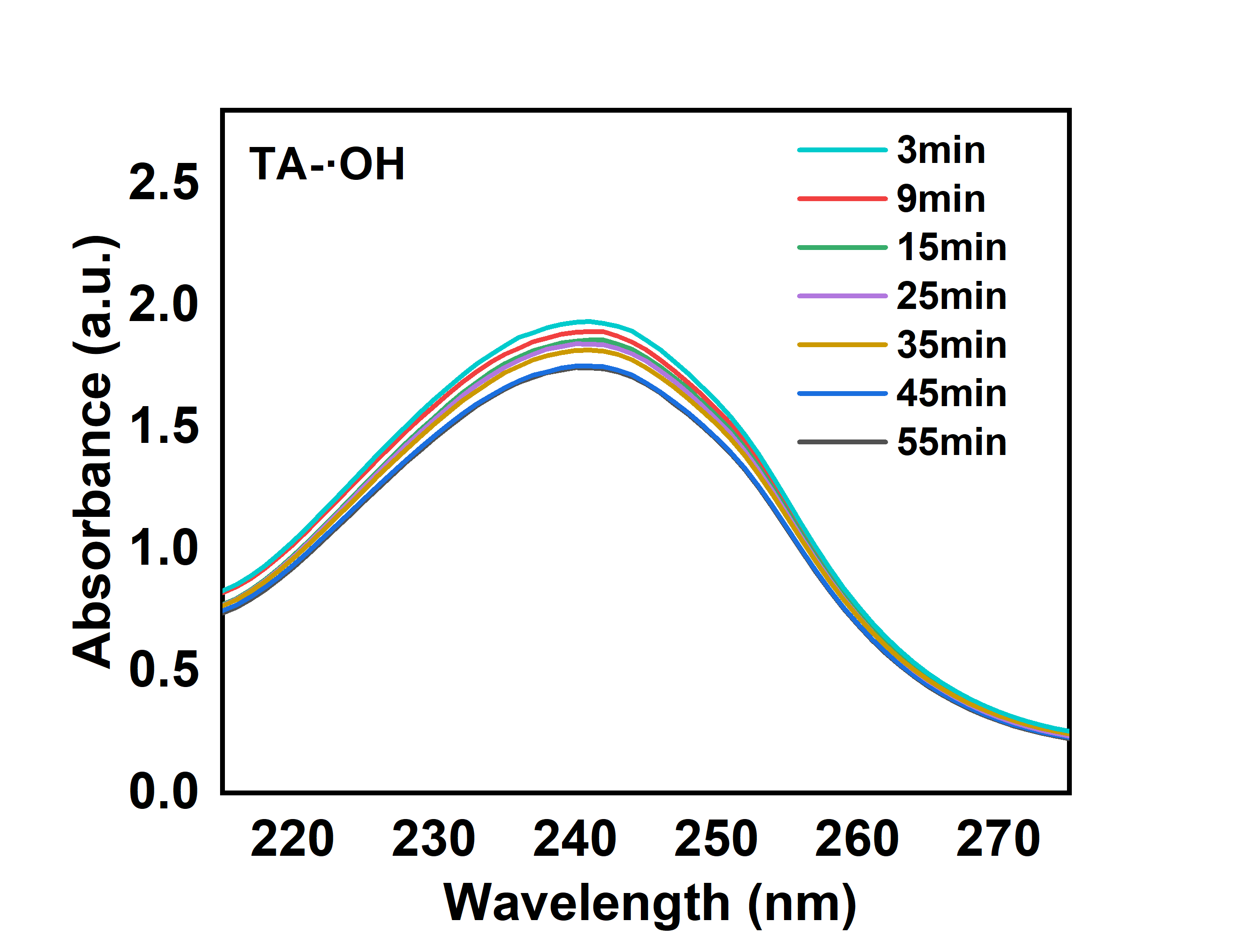


**Figure S32** UV-Vis absorption changes of TA at 240 nm with TroTfb-COF. Reaction conditions: [TA] = 1mM, [Cat.] = 0.25 g L^−1^ in 10 mL water, O_2_ (1 atm), 25 °C, λ = 630 nm.


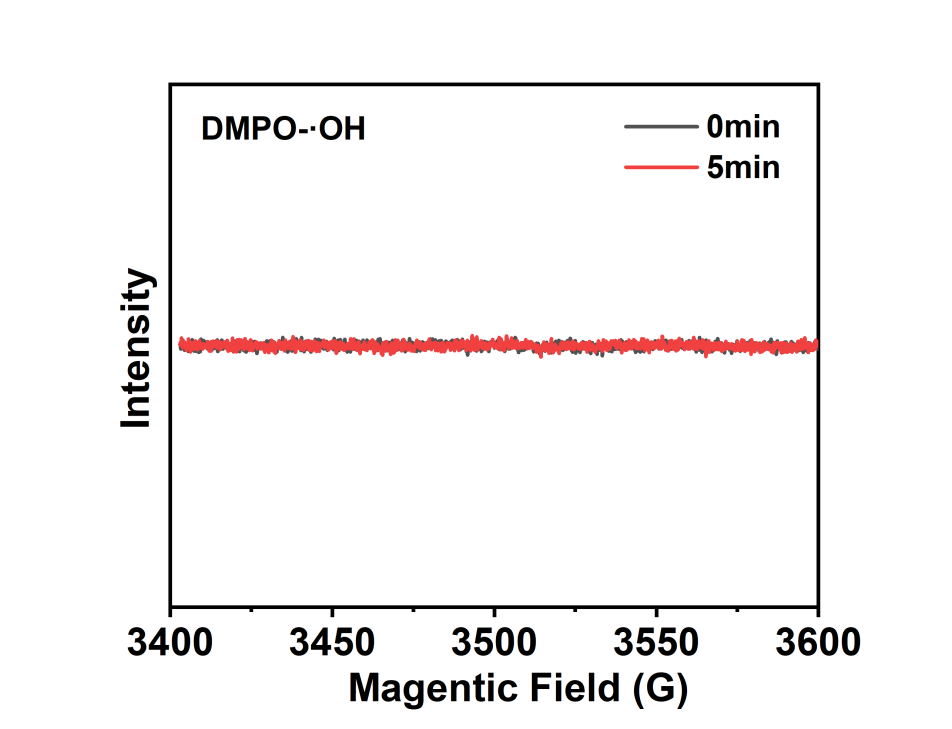


**Figure S33** Spin-trapping EPR spectra of trapping agent DMPO for **^.^**OH.


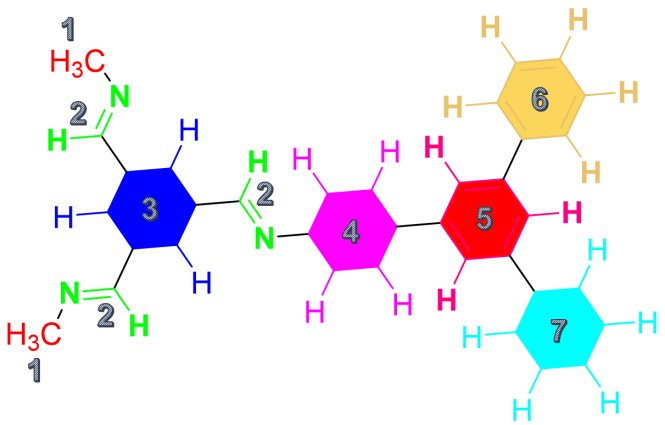


**Figure S34** Schematic diagram of the structural sequence number of TpbTfb-COF.


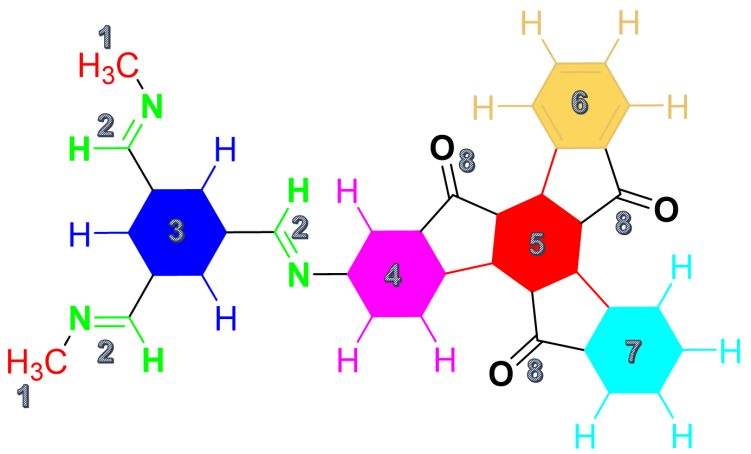


**Figure S35** Schematic diagram of the structural sequence number of TroTfb-COF.

**Section S5. Tables S1-S5**

Table S1. Atomistic coordinates for the reversible AA stacking structure of the TpbTfb-COF

| Atom | x | y | z |
| --- | --- | --- | --- |
| C1 | 0.51180 | 0.97956 | 0.52986 |
| N2 | 0.48007 | 0.89423 | 0.53391 |
| C3 | 0.42060 | 0.71934 | 0.50332 |
| C4 | 0.40227 | 0.84276 | 0.49039 |
| C5 | 0.36823 | 0.75320 | 0.49896 |
| H6 | 0.48771 | 0.76170 | 0.50198 |
| H7 | 0.35701 | 0.86313 | 0.43851 |
| C8 | 0.02259 | 0.53293 | 0.55433 |
| N9 | 0.10836 | 0.58597 | 0.54647 |
| C10 | 0.28242 | 0.70007 | 0.49791 |
| C11 | 0.15819 | 0.55825 | 0.49938 |
| C12 | 0.24817 | 0.61362 | 0.50019 |
| H13 | 0.23989 | 0.72423 | 0.49440 |
| H14 | 0.13600 | 0.49183 | 0.45200 |
| N15 | 0.41666 | 0.52256 | 0.54388 |
| C16 | 0.30093 | 0.58101 | 0.50098 |
| C17 | 0.44370 | 0.60053 | 0.51480 |
| C18 | 0.38767 | 0.63372 | 0.50580 |
| H19 | 0.27625 | 0.51413 | 0.49986 |
| H20 | 0.51055 | 0.64733 | 0.49901 |
| C21 | 0.47040 | 0.49080 | -0.45471 |
| H22 | 0.58156 | 0.59594 | -0.65458 |
| H23 | 1.01972 | 0.42396 | -0.24985 |
| H24 | 1.00363 | 0.63038 | -0.61802 |
| H25 | 0.47039 | 0.13306 | -0.28500 |
| C26 | 0.98281 | 0.45018 | -0.34055 |
| C27 | 0.97280 | 0.56594 | -0.53963 |
| C28 | 0.89681 | 0.40252 | -0.33762 |
| C29 | 0.71504 | 0.29637 | -0.45798 |
| C30 | 0.84646 | 0.43435 | -0.44088 |
| C31 | 0.88700 | 0.51739 | -0.54217 |
| C32 | 0.75481 | 0.38299 | -0.44443 |
| H33 | 0.85061 | 0.54443 | -0.62950 |
| H34 | 0.75273 | 0.26692 | -0.45922 |
| H35 | 0.86801 | 0.33938 | -0.24490 |
| C36 | 0.55358 | 0.53306 | -0.55979 |
| C37 | 0.43702 | 0.40875 | -0.34899 |
| C38 | 0.60051 | 0.49456 | -0.55752 |
| C39 | 0.70515 | 0.41953 | -0.43515 |
| C40 | 0.56762 | 0.41252 | -0.44976 |
| C41 | 0.48462 | 0.37083 | -0.34437 |
| C42 | 0.61834 | 0.37212 | -0.45238 |
| H43 | 0.45687 | 0.30764 | -0.25287 |
| H44 | 0.73472 | 0.48657 | -0.40609 |
| H45 | 0.66388 | 0.52841 | -0.65169 |
| C46 | 0.46772 | 0.01886 | -0.37200 |
| H47 | 0.40426 | -0.01805 | -0.28130 |
| H48 | 0.62893 | -0.00118 | -0.64289 |
| C49 | 0.59472 | 0.02945 | -0.56808 |
| C50 | 0.50547 | 0.10455 | -0.37472 |
| C51 | 0.58120 | 0.28575 | -0.47317 |
| C52 | 0.58813 | 0.15505 | -0.47763 |
| C53 | 0.63142 | 0.11492 | -0.57639 |
| C54 | 0.62837 | 0.24645 | -0.46979 |
| H55 | 0.69524 | 0.15122 | -0.66357 |
| H56 | 0.51393 | 0.24839 | -0.50053 |
| H57 | 0.37256 | 0.37578 | -0.26686 |

Table S2. Atomistic coordinates for the reversible AA stacking structure of the TroTfb-COF

| atom | x | y | z |
| --- | --- | --- | --- |
| N1 | 0.49993 | 0.89374 | 0.50000 |
| C2 | 0.42216 | 0.71301 | 0.50000 |
| C3 | 0.41827 | 0.84795 | 0.50000 |
| C4 | 0.37557 | 0.75576 | 0.50000 |
| H5 | 0.49156 | 0.75083 | 0.50000 |
| C6 | 0.53891 | 0.98238 | 0.50000 |
| C7 | 0.49486 | 0.02797 | 0.50000 |
| H8 | 0.42564 | 0.99755 | 0.50000 |
| H9 | 0.66075 | 0.99057 | 0.50000 |
| C10 | 0.62852 | 0.02770 | 0.50000 |
| C11 | 0.54151 | 0.11525 | 0.50000 |
| C12 | 0.58469 | 0.26108 | 0.50000 |
| C13 | 0.63129 | 0.16089 | 0.50000 |
| C14 | 0.67552 | 0.11630 | 0.50000 |
| C15 | 0.65885 | 0.25178 | 0.50000 |
| H16 | 0.74476 | 0.15028 | 0.50000 |
| C17 | 0.82516 | 0.33305 | 0.50000 |
| O18 | 0.43242 | 0.15438 | 0.50000 |
| H19 | 0.49932 | 0.62462 | 0.50000 |

Table S3. Photocatalytic synthesis of benzimidazole by cascade reaction over TroTfb-COF under different conditions^a^


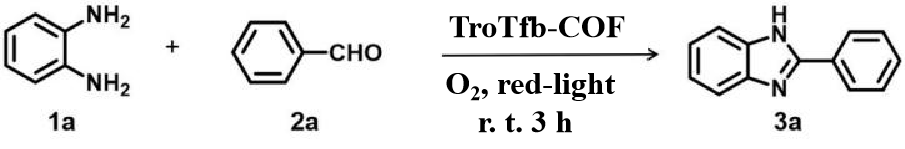


| **Entry** | **Catalyst** | **Solvent** | **Time (h)** | **Atmosphere** | **Light** | **Yield^b^ (%)** |
| --- | --- | --- | --- | --- | --- | --- |
| 1 | 3mg | DMF | 1 | O_2_ | red | 55 |
| 2 | 3mg | MeOH | 1 | O_2_ | red | 68 |
| 3 | 3mg | EtOH | 1 | O_2_ | red | 75 |
| 4^d^ | 3mg | EtOH | 1 | N_2_ | red | 0 |
| 5 | - | EtOH | 1 | O_2_ | red | 10 |
| 6 | 1mg | EtOH | 1 | O_2_ | red | 48 |
| 7 | 2mg | EtOH | 1 | O_2_ | red | 53 |
| 8^d^ | 3mg | EtOH | 1 | O_2_ | black | 0 |
| 9^e^ | 3mg | EtOH | 1 | O_2_ | blue | 64 |
| 10 | 3mg | EtOH | 2 | O_2_ | red | 90 |
| 11 | 3mg | EtOH | 3 | O_2_ | red | 98 |

^a^ Reaction conditions: 1a (0.25 mmol), 2a (0.25 mmol), photocatalyst, solvent (3 mL), O_2_ atmosphere, red-LED light (20 w, 630 nm), room temperature. ^b^ Determined by ^1^H NMR. ^c^ In N_2_ atmosphere. ^d^ In darkness. ^e^ Blue LED (30 w, 455 nm).

**Table S4**. Efficiency comparison of TroTfb-COF and other reported photocatalysts for singlet oxygen production

| Test method | Catalyst | ^1^O_2_ concentration  (μM) | Time  (s) | dosage  (g L^-1^) | ^1^O_2_ yield  (μM s^−1^) | Ref |
| --- | --- | --- | --- | --- | --- | --- |
| DPBF | CoSAC-NG | 20.67 | 600 | 0.005 | 0.03 | 14 |
| DPBF | Zn_4_Co_1_-C | 640 | 600 | 0.1 | 0.1 | 15 |
| FFA | Vis/TiFeAS | 730 | 600 | 0.2 | 1.2 | 16 |
| DPBF | NG | 3.76 | 600 | 0.005 | 0.006 | 14 |
| DPBF  DPBF | DAQ-COF  TroTfb-COF | 890  634 | 60  60 | 0.25  0.25 | 14.8  10.6 | 17  This work |

^−^Table S5. Investigation of the photocatalytic oxidation of sulfides (1a) using various quenchers, and TroTfb-COF as the catalyst

| **Scavenger** | **Species quenched** | **Yield (%)** |
| --- | --- | --- |
| KI | h^+^ | 36 |
| DDQ | e^-^ | 11 |
| *p*-BQ | O_2_^-^ | 69 |
| DBACO | ^1^O_2_ | 3 |
| *i*-PrOH | ^·-^OH | 89 |

**Section S6. References**

1. M. Liu, J. Liu, J. Li, Z. Zhao, K. Zhou, Y. Li, P. He, J. Wu, Z. Bao, Q. Yang, Y. Yang, Q. Ren, Z. Zhang, *J. Am. Chem. Soc*. 2023, ***145***, 9198-9206.
2. Y. Zhao, X. Xu, K. Zhang, Z. Li, H. Wang, Y. Zhao, J. Qiu, J. Wang, *ACS Catal*. 2024, ***14***, 3556-3564.
3. S. Li, C. Liu, W. Lv, G. Liu, *Environ. Sci. Technol*. 2022, ***56***, 4980-4987.

[4] G. Kresse, J. Furthmüller, *Phys. Rev., B Condens. Matter* 1996, ***54***, 11169-11186.

[5] G. Kresse, D. Joubert, *Phys. Rev. B* 1999, ***59***, 1758-1775.

[6] J. P. Perdew, K. Burke, M. Ernzerhof, *Phys. Rev. Lett*. 1997, **78**, 1396-1396.

[7] J. P. Perdew, M. Ernzerhof, K. Burke, *J. Chem. Phys. (USA)* 1996, ***105***, 9982-9985.

[8] H. J. Monkhorst, J. D. Pack, *Phys. Rev. B* 1976, ***13***, 5188-5192.

[9] M. J. Frisch, G. W. Trucks, H. B. Schlegel, G. E. Scuseria, M. A. Robb, J. R. Cheeseman, G. Scalmani, V. Barone, B. Mennucci, G. A. Petersson, H. Nakatsuji, M. Caricato, X. Li, H. P. Hratchian, A. F. Izmaylov, J. Bloino, G. Zheng, J. L. Sonnenberg, M. Had, D. J. Fox, *Revision A*. 2009, 01.

[10] L. Li, Z. Zhou, L. Li, Z. Zhuang, J. Bi, J. Chen, Y. Yu, J. Yu, *ACS Sustainable Chem. Eng*. 2019, ***7***, 18574-18581.

[11] H. Ben, G. Yan, H. Liu, C. Ling, Y. Fan, X. Zhang, *Adv. Funct. Mater*. 2021, ***32***, 2104519.

[12] W. Humphrey, A. Dalke, K. Schulten, *J. Molec. Graphics*. 1996, ***14***, 33-38

[13] T. Lu, F. W. Chen, *J. Comput. Chem*. 2012, ***33***, 580-592.

[14] H. Zhao, J. Song, P. Lu, Y. Mu, *Chem. Eng.* J. 2023, ***456***, 141045.

[15] Y. Yao, C. Wang, X. Yan, H. Zhang, C. Xiao, J. Qi, Z. Zhu, Y. Zhou, X. Sun, X. Duan, J. Li. *Environ. Sci. Technol*. 2022, ***56***, 8833-8843.

[16] F. Mo, C. Song, Q. Zhou, W. Xue, S. Ouyang, Q. Wang, Z. Hou, S. Wang, J. Wang. *Proc. Natl. Acad. Sci*. 2023, ***120***, 306 e2300281120.

[17] Y. Tao, Y. Hou, H. Yang, Z. Gong, J.Yu, H. Zhong, Q. Fu, J. Wang, F. Zhu, G. Ouyang, *Proc. Natl. Acad. Sci*. 2024, 121, e2401175121.

**Section S7** ^1^H NMR spectrum of the corresponding products


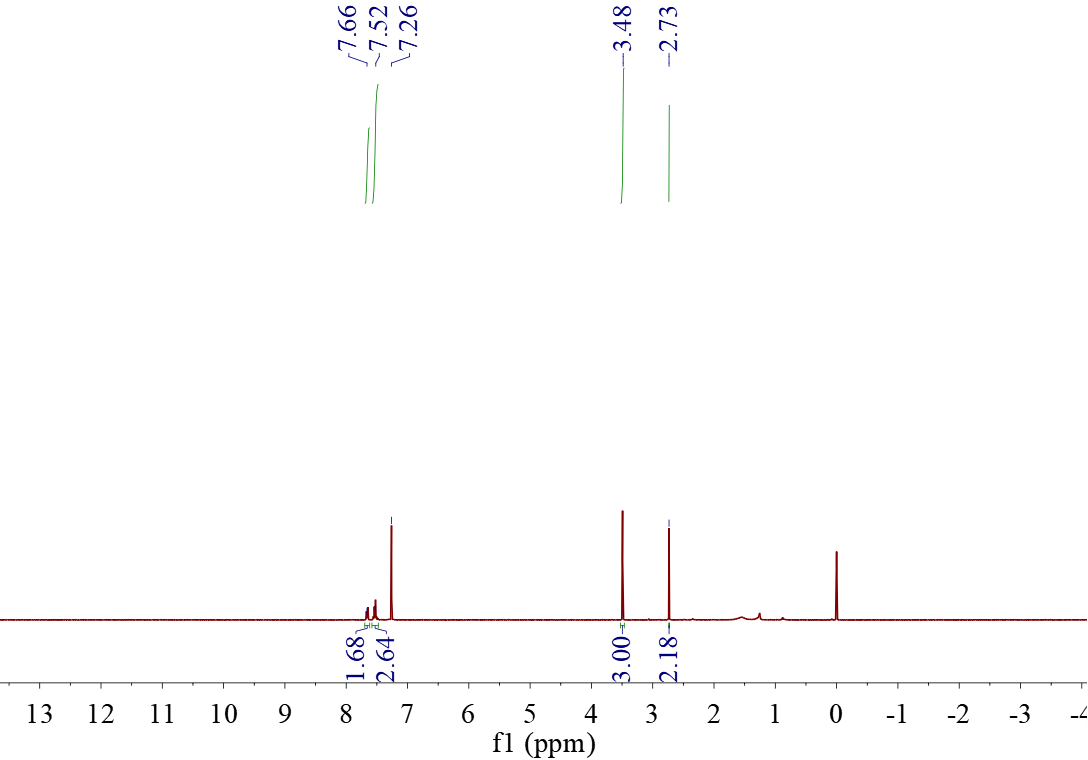


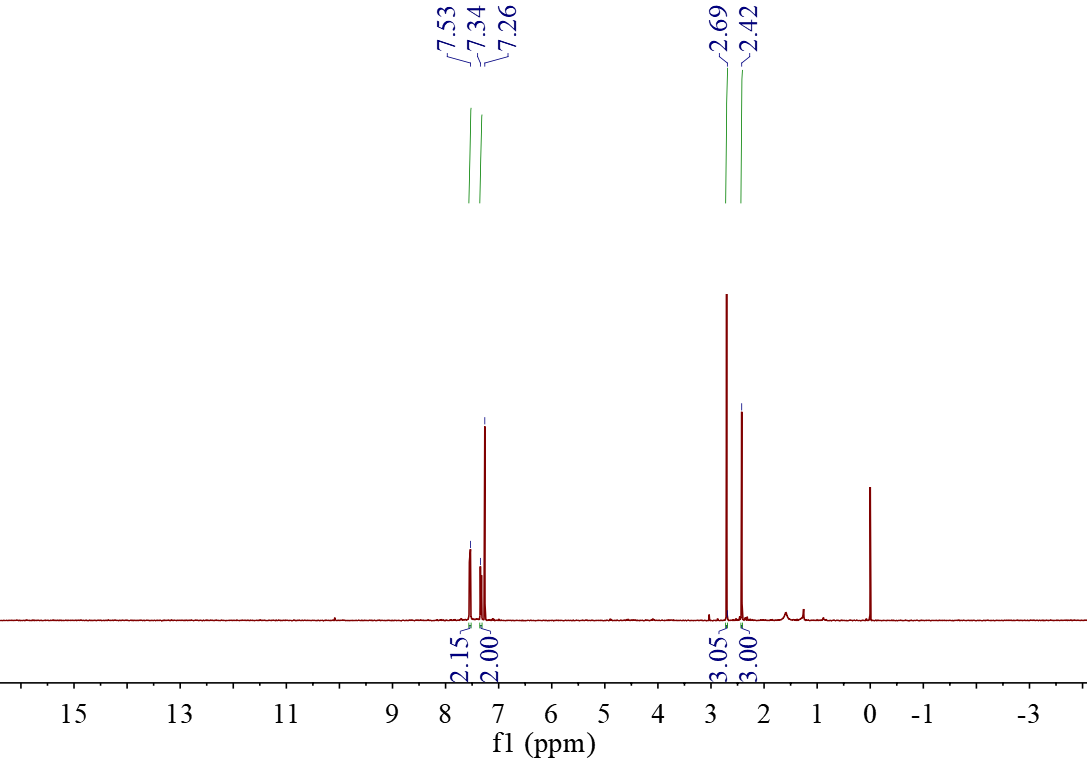


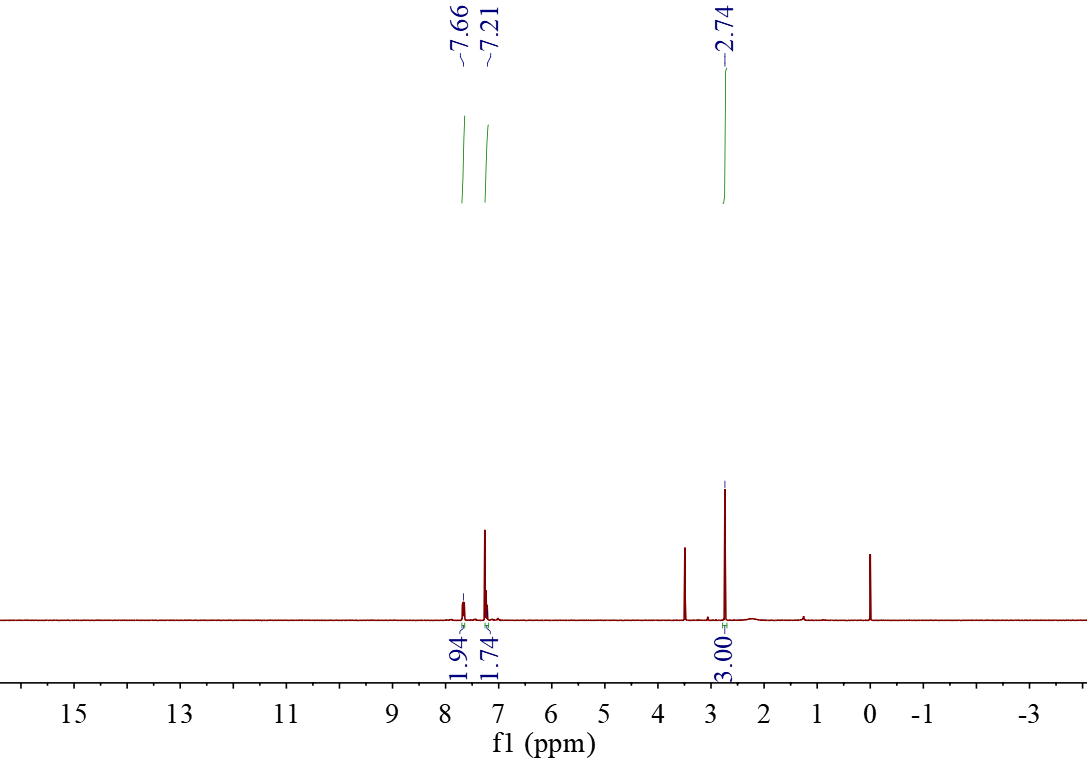


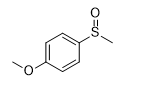


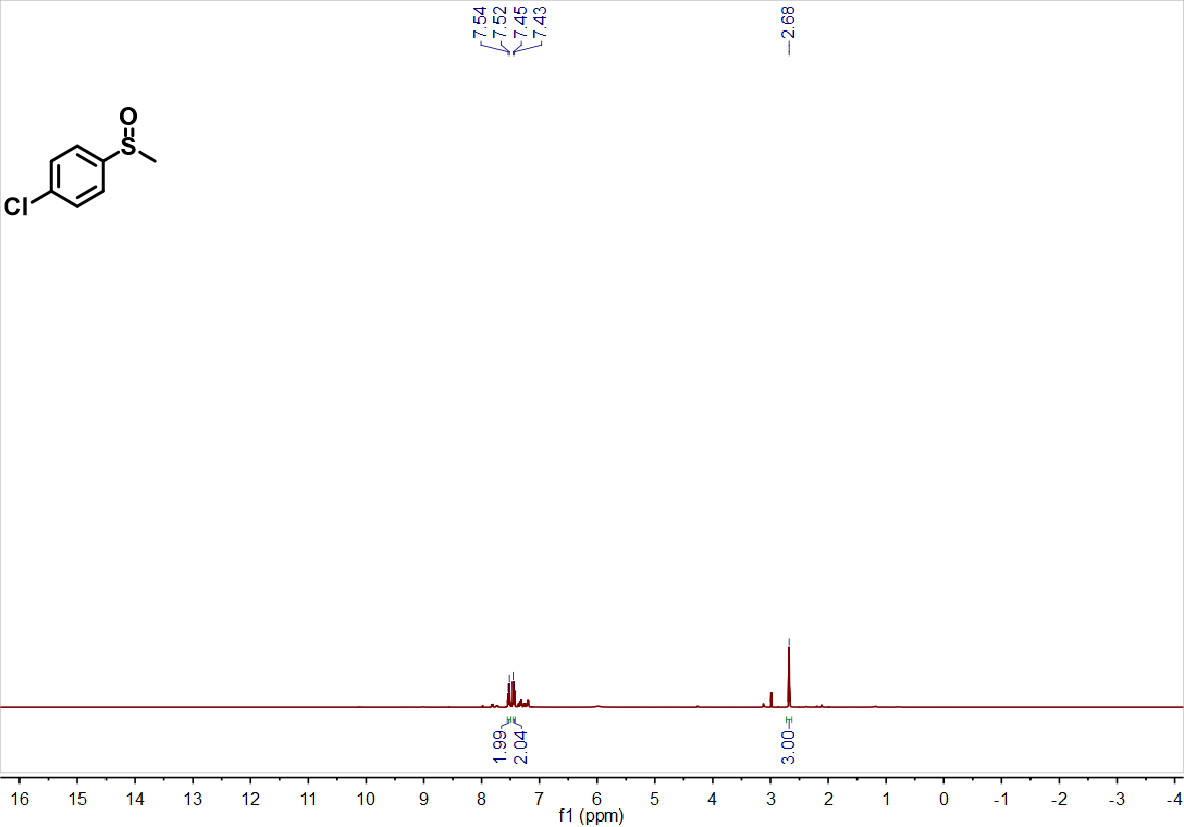


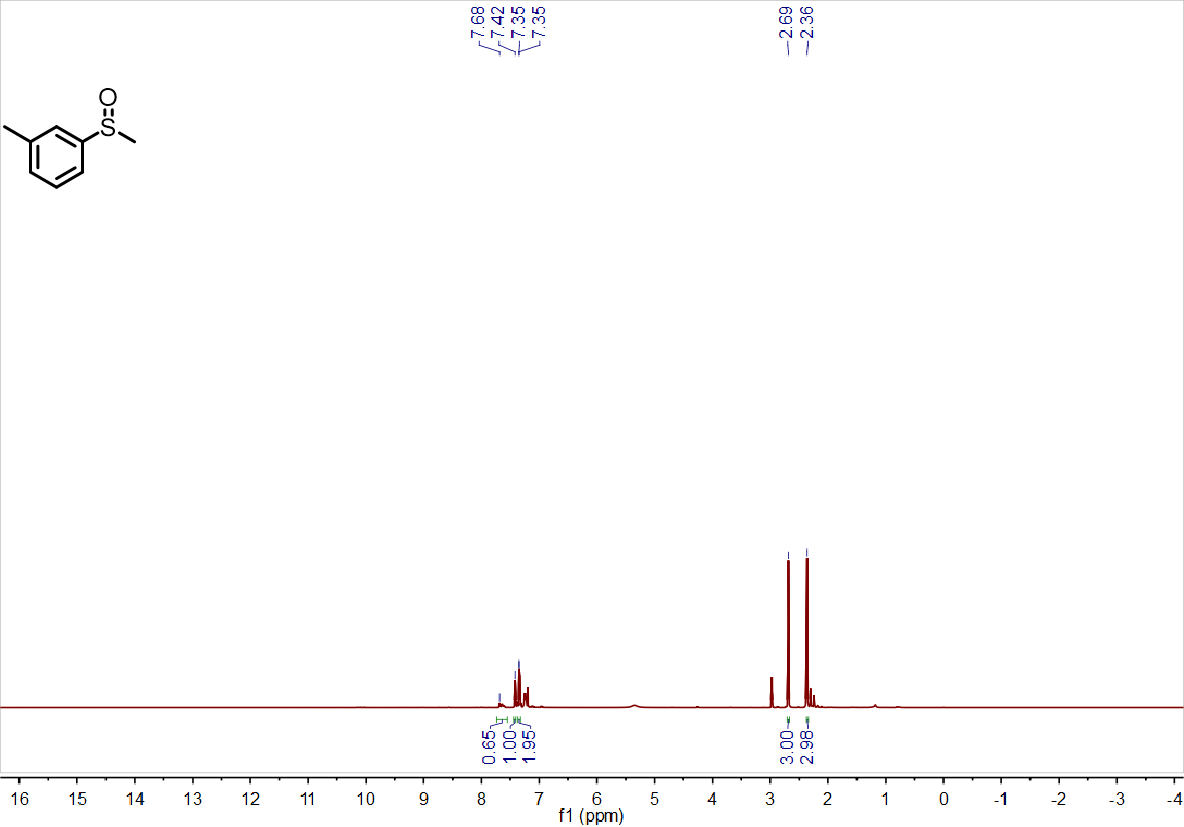


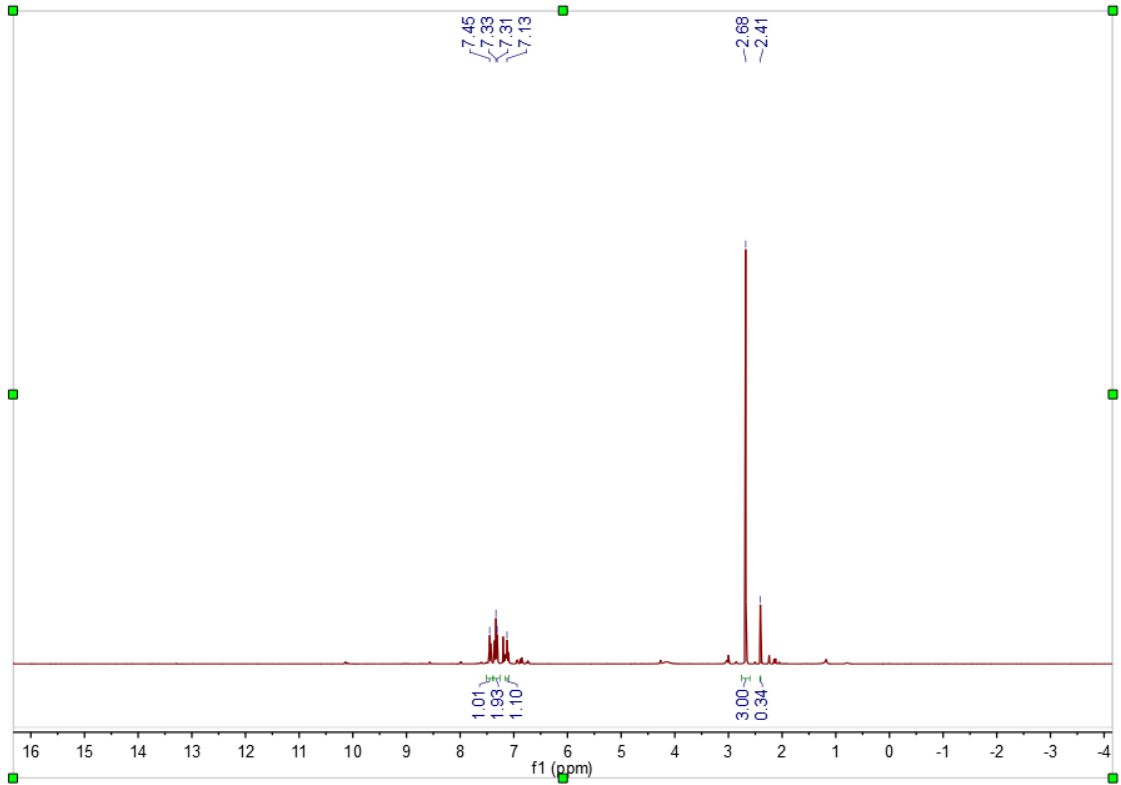


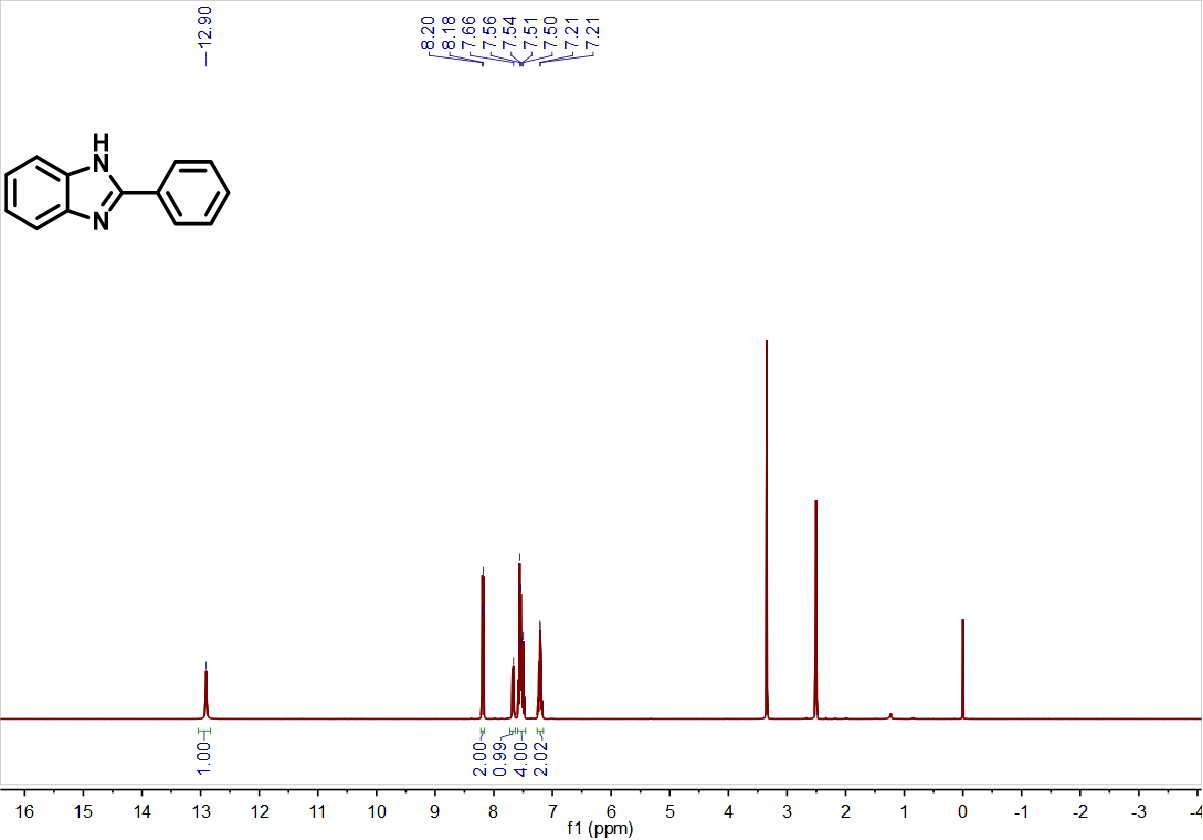


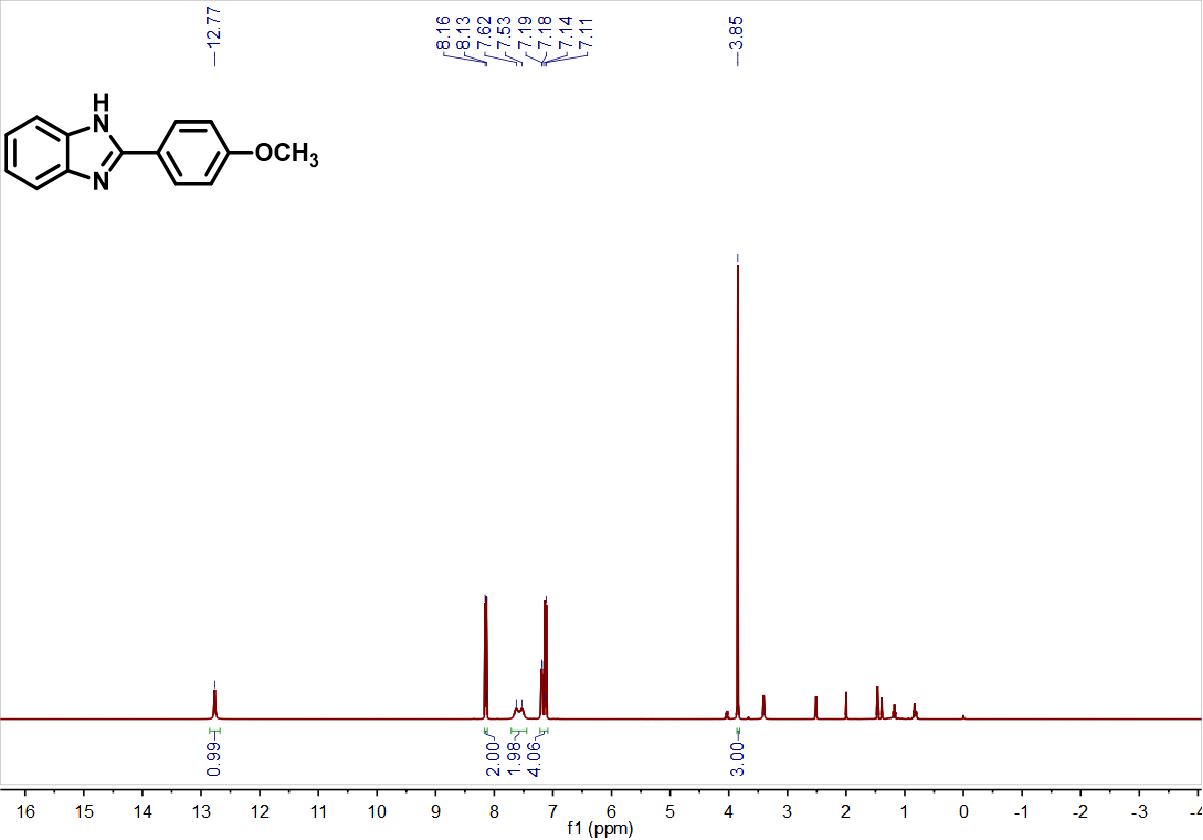


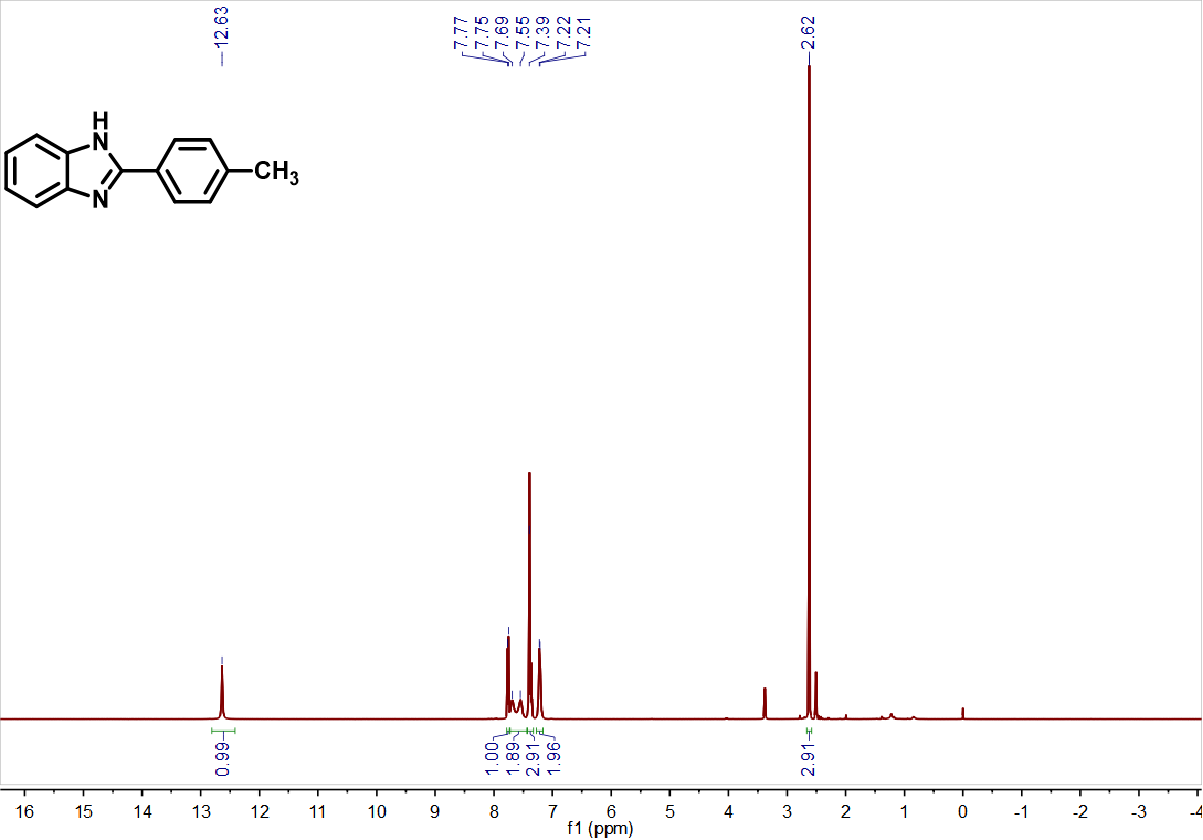


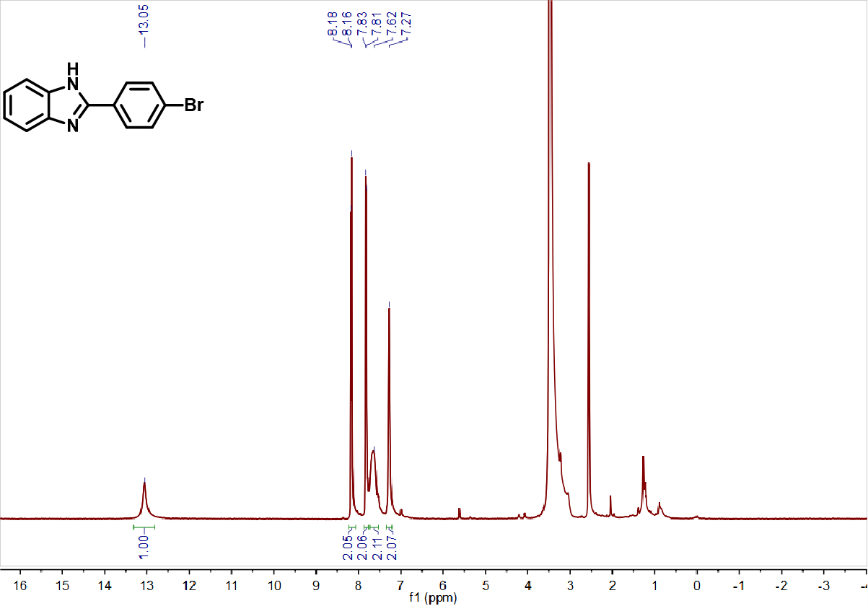


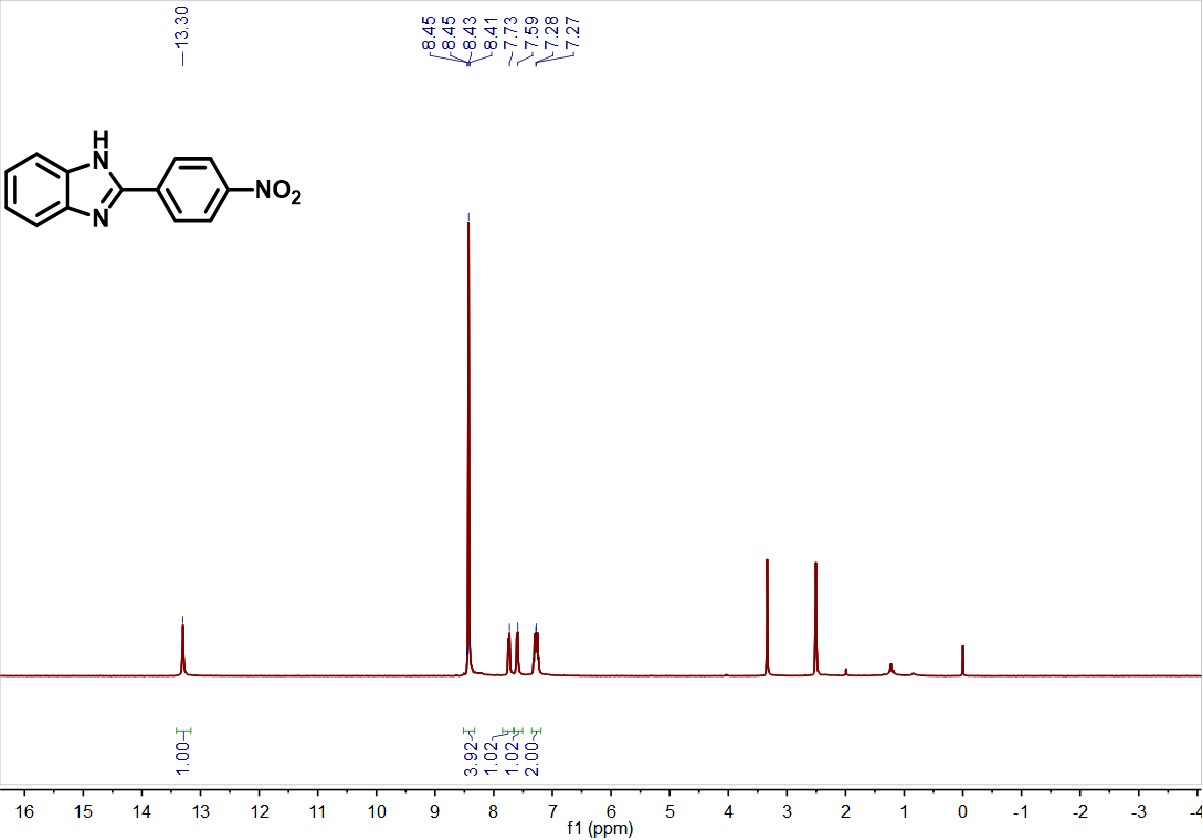


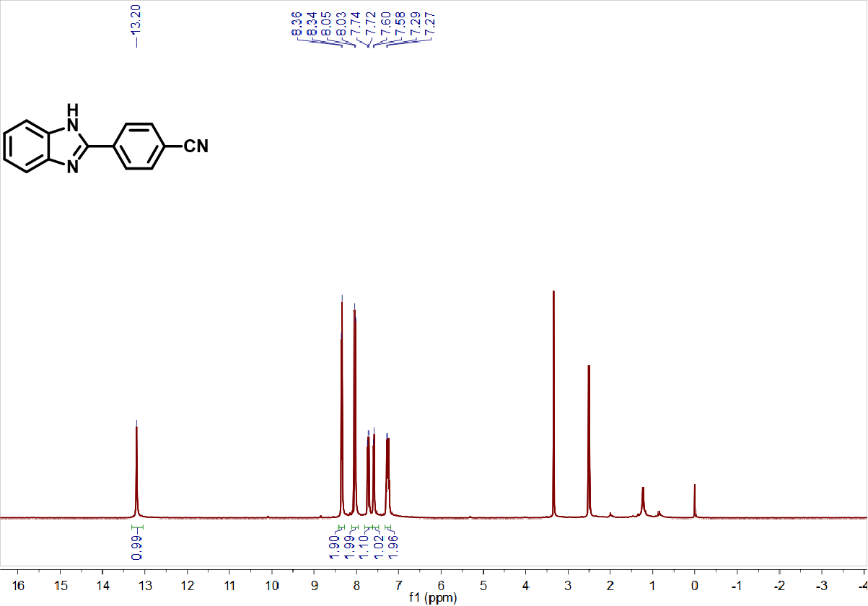


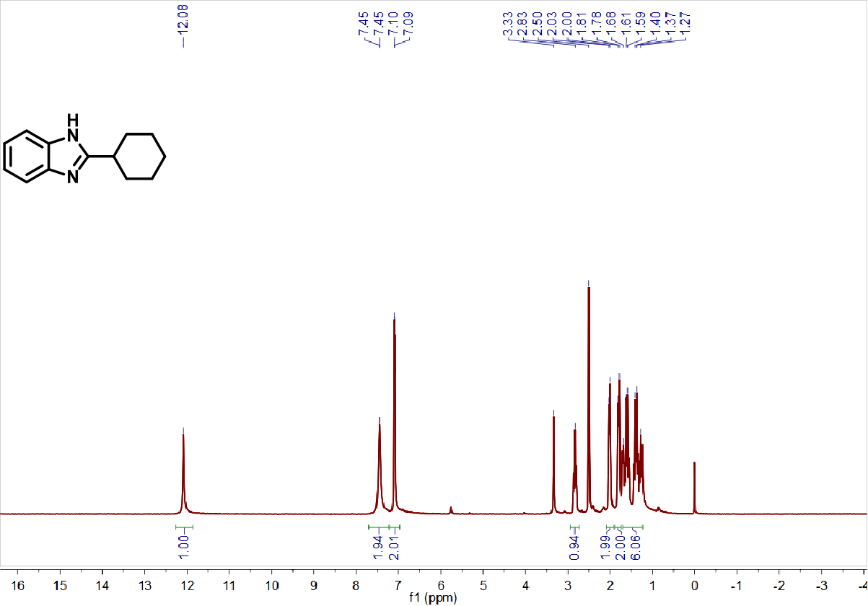


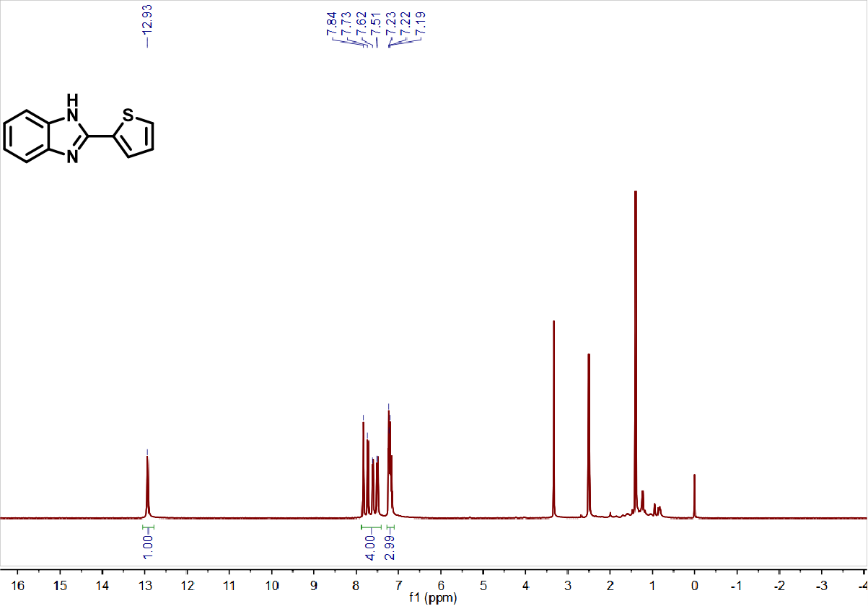


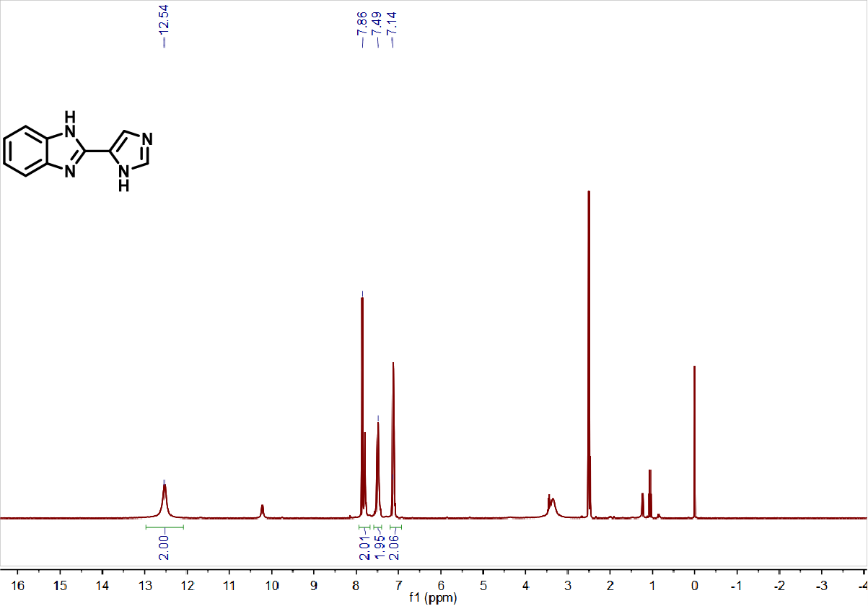


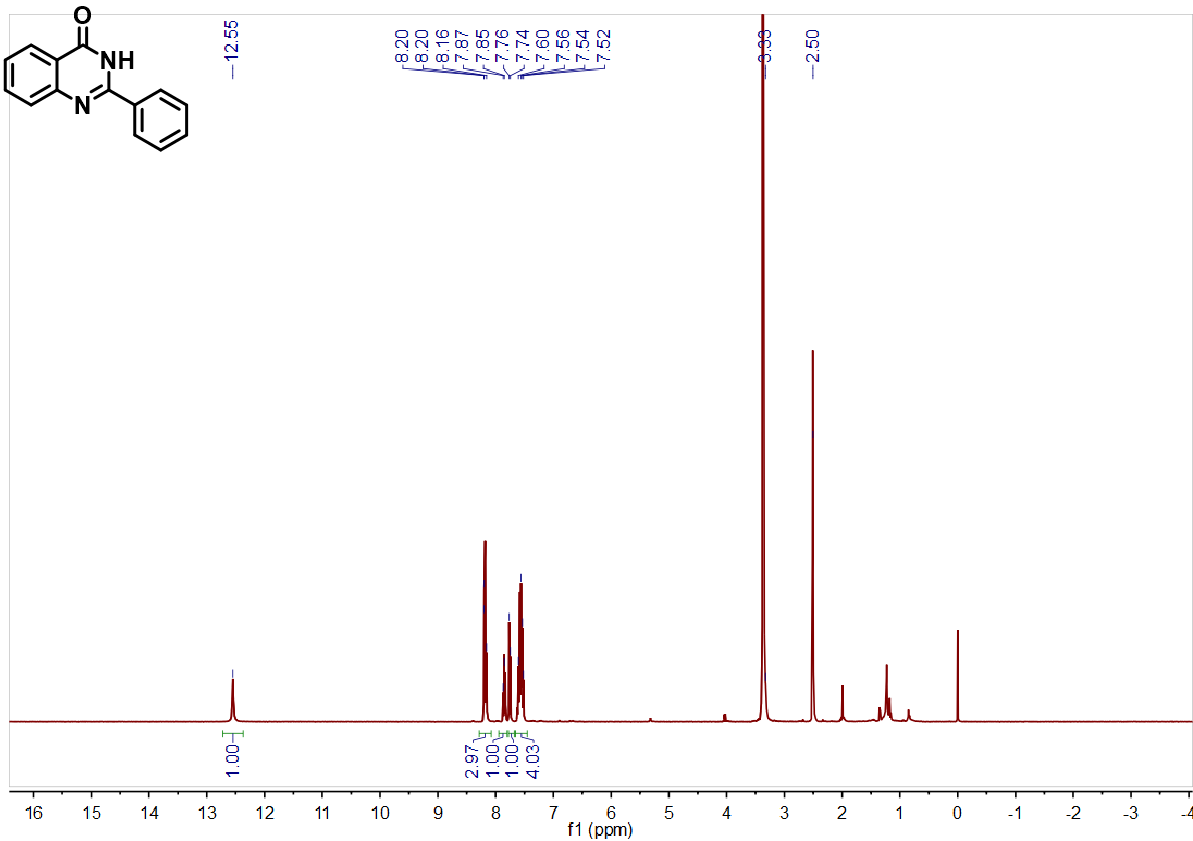


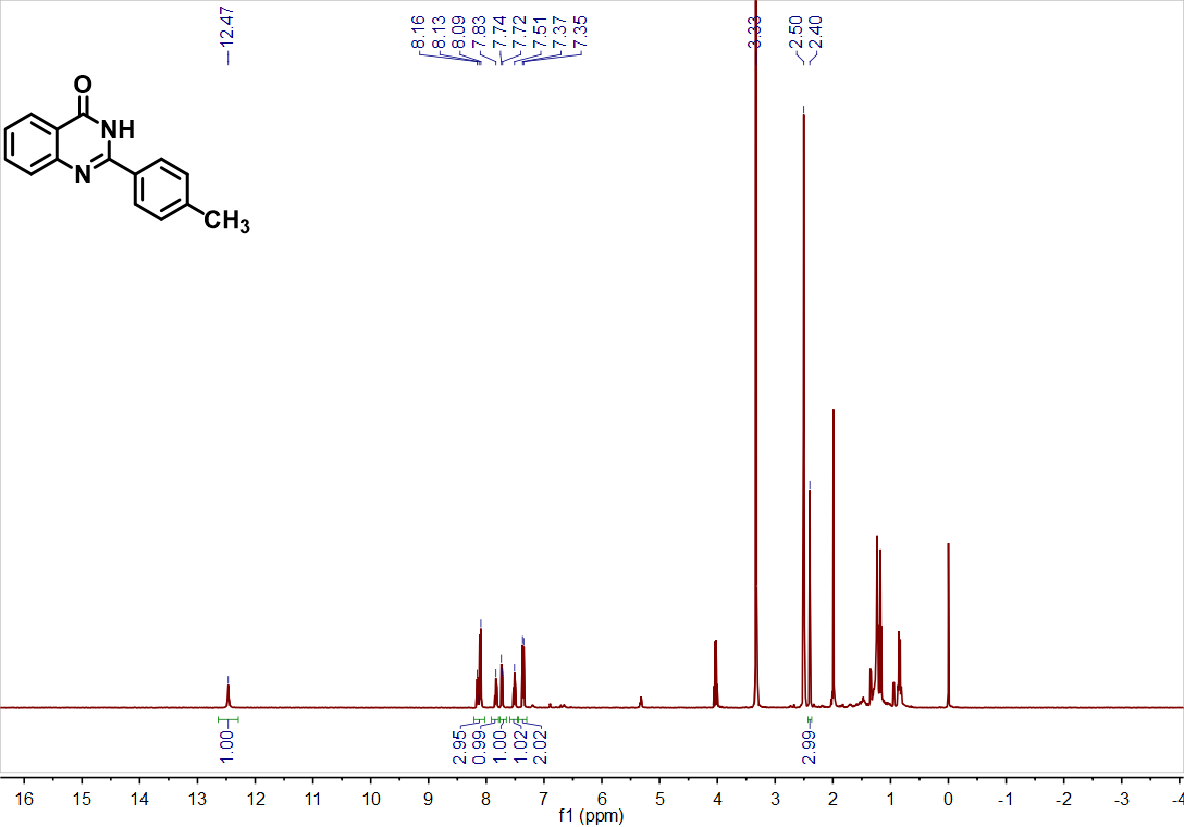


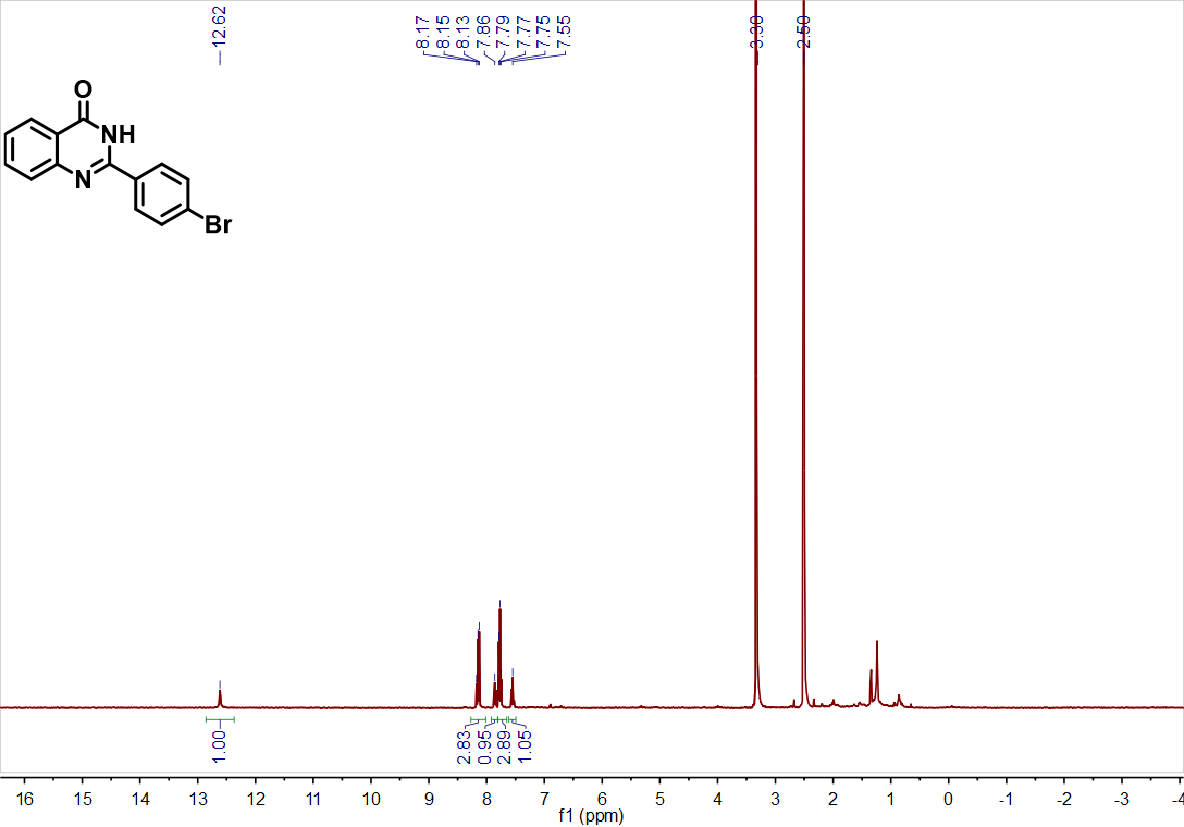


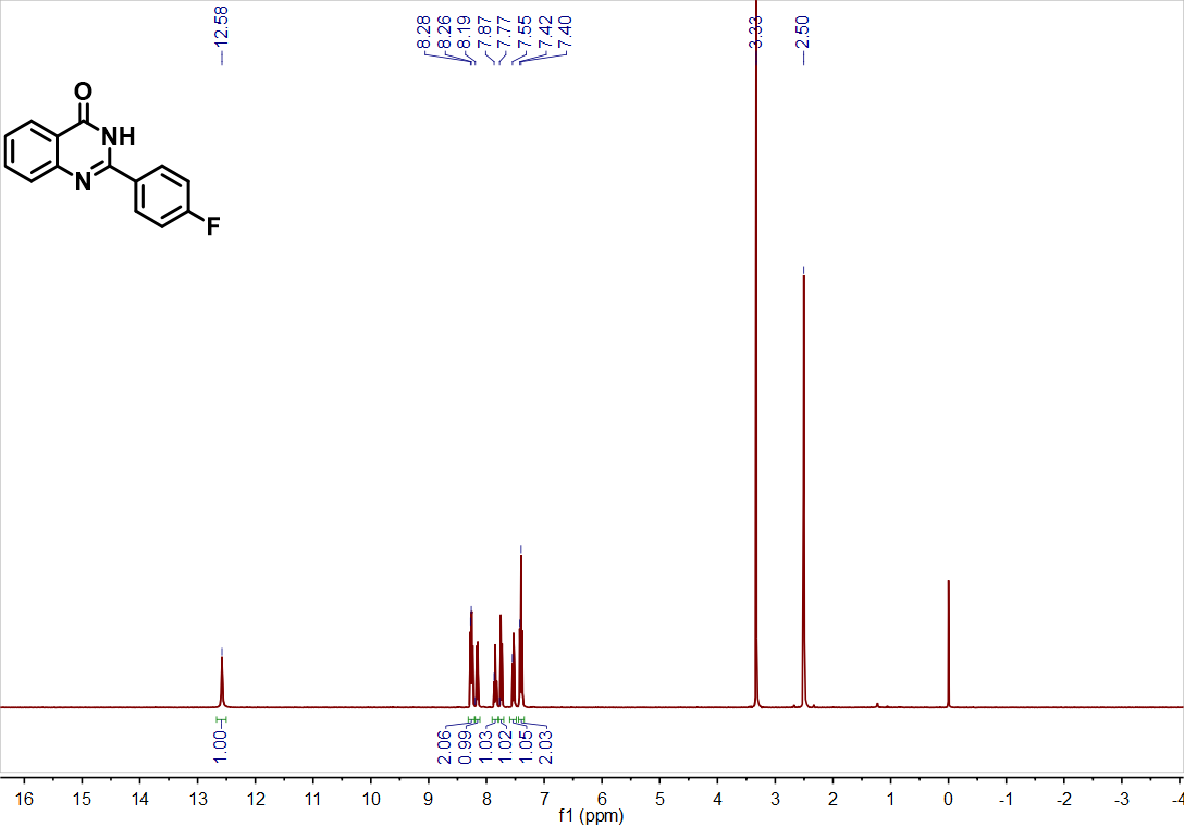

Supplement: Supplementary file 1 — Supporting Information [file ANIE-64-e202508078-s001.docx]
